# Active Compounds and Targets of Yuanzhi Powder in Treating Alzheimer's Disease and Its Relationship with Immune Infiltration Based on HPLC Fingerprint and Network Pharmacology

**DOI:** 10.1155/2022/3389180

**Published:** 2022-07-15

**Authors:** Qingsong Liu, Shaofeng Wang, Yanwei Hao, Jiaxin Li, Wei Li, Yi Zhang, Bin Li

**Affiliations:** ^1^Department of Gastroenterology, Hospital of Chengdu University of Traditional Chinese Medicine, Chengdu 610072, China; ^2^School of Clinical Medicine, Chengdu University of Traditional Chinese Medicine, Chengdu 610075, China; ^3^Department of Geriatrics, Hospital of Chengdu University of Traditional Chinese Medicine, Chengdu 610072, China

## Abstract

**Background:**

Yuanzhi powder (YZP) has been extensively investigated as a natural prescription with therapeutic benefits for Alzheimer's disease (AD). However, its active compounds and underlying immune mechanism for treating AD are still unclear. This study aimed to investigate the immune mechanism of YZP against AD through high-performance liquid chromatography (HPLC)-based network pharmacology and gene chip technology.

**Methods:**

Active components of YZP were obtained from HPLC and public databases. Subsequently, GSE5281, GSE28146, GSE29378, and GSE97760 from the Gene Expression Omnibus (GEO) database were downloaded to extract AD difference genes (DEGs). The active components-targets network and protein interaction network were then constructed by Cytoscape. The biological processes and signaling pathways, which implicate the targets of YZP for AD, were analyzed using the ClueGo Cytoscape plug-in. Molecular docking experiments were performed to verify the affinity of targets and ligands. Ultimately, the link between the hub genes and immune cell infiltration was assessed via CIBERSORT.

**Results:**

83 YZP active compounds and 641 DEGs associated with AD, including quercetin, berberine, 3,6′-disinapoylsucrose, coptisine, and palmatine, were evaluated. We showed that FOS, CCL2, and GJA1 were the core targets and that the gap junction is an essential signaling pathway in YZP for AD. Furthermore, the AD group had a higher infiltration level of naïve B cells and resting CD4 memory T cells, as determined by the CIBERSORT. Notably, the immune cells-targets network demonstrates that GJA1 and GRM1 are intimately related to naïve B cells and plasma cells.

**Conclusions:**

YZP may help treat AD by targeting proteins with key active compounds to regulate naïve B cells and plasma cells. Our results demonstrate a new immune mechanism for treating AD with YZP.

## 1. Introduction

Alzheimer's disease (AD) is a progressive neurodegenerative disease with insidious onset. According to a US study, by 2060. about 15 million people will be affected by AD [1]. Thus, AD has emerged as a severe threat to the aging population's health and quality of life. AD currently has no cure and is highly disabling and irreversible. Patients generally die within 5–12 years of the beginning of symptoms [2]. Natural products have offered a significant deal of promise for treating AD for many years due to their many components [3]. Therefore, natural products provide an attractive approach to prevent and treat AD.

Yuanzhi powder (YZP), derived from the General Record of Shengji (*shenjizonglu*), written during the Song Dynasty in China, has been used in therapeutic preparation for almost a century where experimental research has shown its efficacy. Studies show that YZP can suppress amyloid *β*-protein (A*β*) aggregation in AD [4]. YZP is composed of herbs like Polygala tenuifolia Willd (*Yuanzhi*), Acorus tatarinowii (*Shichangpu*), Coptis chinensis Franch (*Huanglian*), Poria (*Fuling*), and Ginseng Radix et Rhizoma (*Renshen*). *Yuanzhi*, *Huanglian*, and *Renshen* have been widely investigated for their neuroprotective properties, such as anti-A*β* aggregation, antitau protein, strengthening the central cholinergic system, and encouraging neuronal proliferation in AD [5–7]. *Shichangpu* may have anti-inflammatory and neuroprotective properties [8]. *Fuling* contributes to AD treatment by controlling gut flora and decreasing inflammation [9]. Despite the popularity of YZP in AD replacement therapy, the pharmacological effects and its immune mechanism on AD remain unclear.

There is mounting evidence indicating that the etiology of AD is intimately linked to immunological reactions [10]. The binding of AD-related proteins and receptors on microglia may cause the progression of the disease [11]. Studies have shown that Chinese herbal herbs in YZP can improve immune function. For example, *Yuanzhi, Shichangpu*, and *Fuling* can act as immune enhancers [12–14]. *Huanglian* possesses anti-inflammatory and immunomodulatory properties [15, 16]. *Renshen* can boost macrophage phagocytic capability and NK cell activity [17]. However, the mechanism of YZP for treating AD by immune cell regulation remains largely unknown. Therefore, this study aims to characterize the active compounds and targets of YZP in the treatment of AD and their relationship with immune infiltration. Our results demonstrate the immune mechanism of YZP in treating AD, thus providing direction in AD research.

## 2. Methods

### 2.1. Instruments and Chemicals

A high-performance liquid chromatography (HPLC, Shimadzu LC-2030) model was used.

Kunshan Ultrasonic Instrument Co., Ltd. supplied the BJ-150 multi-function crusher. Mettler Toledo Co., Ltd. offered the MS105DU electronic analytical balance. KQ-500DE ultrasonic cleaning machine was from Baijie Electric Appliance Co., Ltd., Deqing County, Zhejiang.

*Yuanzhi* (Shanxi Province, batch No. 191001), *Shichangpu* (Guangxi Province, batch No. 200301), *Huanglian* (Sichuan Province, batch No. 200101), *Fuling* (Anhui Province, batch No. 200201), *Renshen* (Jilin Province, batch No. 191101) came from different real estate area. 3,6′-disinapoylsucrose, Coptisine, Palmatine, and Berberine were purchased from the China Institute of Food and Drug Verification and Research (Beijing, China). Chromatographic grade acetonitrile (ACN) was purchased from Shanghai Petrology Experiment Technology Co., Ltd. (Shanghai, China). Formic acid was from the Chinese Pharmaceutical Group Chemical Reagent Co., Ltd. (Beijing, China). Milli-Q system (Aquapro, USA) was used to purify deionized water.

### 2.2. Preparation of YZP Solution

50 g of coarse YZP powder was soaked in 500 mL distilled water for 30 minutes. Subsequently, these powders were refluxed at 100°C twice (for 20 min each time), and then filtered through a 300 mm sieve. Under lower pressure, the extract was filtered and concentrated. The final sample solution was created by dissolving 4 g of the extract in methanol. The reference solution was prepared by weighing and adding appropriate amounts of 3,6′-disinapoylsucrose, palmatine, coptisine, and berberine, to methanol (0.04 mg 3,6′-disinapoylsucrose, 0.04 mg palmatine, 0.04 mg coptisine, and 0.08 mg berberine per 1 ml).

### 2.3. Chromatographic Conditions

The chromatographic column used was Durashell C18 (250 mm × 4.6 mm, 5 *μ*m). Acetonitrile was used as Mobile phase A, and 0.02 mol/L potassium dihydrogen phosphate solution was used as mobile phase B. The following gradient was used for the analysis: 0–5 min, 12%–15% A; 5–15 min, 15%–18% A; 15–40 min, 18%–25% A; 40–90 min, 25–50% A. Wavelength of 246 nm was used to detect the signal, and the flow rate was 1 mL/min. A volume of 10 *μ*L was injected into the column with a temperature of 40°C.

### 2.4. Identification of Compounds and Target Prediction

All putative target genes of the chemical component quantified by HPLC were predicted using Swiss Target Prediction (https://www.swisstargetprediction.ch/). The analytical platform used structural similarities of known components to determine the potential target genes of the sample.

### 2.5. The Active Compounds of YZP from Public Databases

Traditional Chinese Medicine Database and Analysis Platform (TCMSP, https://tcmsp-e.com/) retrieved the active compounds of *Renshen*, *Fuling*, *Shichangpu*, and *Huanglian* and defined the screening criteria as oral bioavailability (OB) ≥30%, drug-like properties (DL) ≥0.18. As the chemical components of *Yuanzhi* were not included in TCMSP, the components were examined in Traditional Chinese Medicines Integrated Database (TCMID, https://47.100.169.139/tcmid/) and HERB (https://herb.ac.cn/). The target protein name was converted into a gene name using the UniProt database (https://www.uniprot.org/uploadlists/).

### 2.6. Screening of AD Differential Genes

The AD-related data files were downloaded from the Gene Expression Omnibus public database (GEO) (https://www.ncbi.nlm.nih.gov/geo/), and the clinical information of the data sets is shown in Table 1. The sva package removed batch effects and merged different data sets. Principal component analysis (PCA) graphs were drawn by package ggord. The limma package was used to perform differential expression analysis with *P* < 0.05 and |log2FC| ＞ 0.584 as the screening criteria, and the volcano map was drawn. The 50 upregulated and 50 downregulated genes with the most significant changes were visualized by drawing heatmaps using the pheatmap package. All packages are run via R software.

### 2.7. Construction of Protein Interaction Network

A Venn diagram was drawn at the junction of YZP targets and AD differential genes to identify AD-related YZP targets. The AD-associated YZP targets were imported into the STRING database (https://cn.string-db.org/). The free nodes were hidden, and the TSV file was obtained. Then Cytoscape version 3.9.0 was imported to generate the protein-protein interaction (PPI) network.

### 2.8. Components-Targets Network

Activated components and AD-related YZP targets were imported into the Cytoscape software, then used to form the components-targets network.

### 2.9. Enrichment Analysis

The ClueGO V2.5.8 (https://apps.cytoscape.org/apps/cluego) plug-in of Cytoscape was used to perform Gene Ontology (GO) biological process and the Kyoto Encyclopedia of Genes and Genomes (KEGG) pathway enrichment analysis on the intersection target. “GO_BiologicalProcess-EBI-UniProt-GOA-ACAP-ARAP_13.05.2021_00h00&quot” and “Genes in KEGG_13.12.2021: 8100” were used to set the parameters *P* < 0.05, Kappa Score Threshold ≥0.35, and the rest of the parameters were the default for GO and KEGG enrichment analysis.

### 2.10. Molecular Docking

The three-dimensional structure of the ligands was created in ChemOffice and stored in the energy-efficient MOL2 format. The Protein Data Bank (PDB) (https://www.rcsb.org/) was used to get the 3D models of the targets. Hydrogen and water were removed by using PyMOL software. We used AutoDock Vina to blind dock ligands and targets. The three coordinates of the grid box were changed to cover the whole protein. AutoDock Vina computed all potential binding residues and displayed the parameter comprising the residues, binding energy, and cluster.

### 2.11. Construction of an Immunoregulatory Network of YZP in AD Treatment

The CIBERSORT is commonly used to assess the microenvironment's immune cell type. The program uses linear support vector regression to deconvolute the expression matrix of immune cell subtypes. The method determines the content of each immune cell subgroup and generates a list of 22 immune cell subtypes' gene expression characteristics. R software was used to quantify the infiltration ratio of 22 immune cells in AD samples. In addition, Spearman correlation analysis was performed on immune cells and AD-related YZP targets. Results obtained from the correlation analysis were imported into Cytoscape to construct an immunoregulatory network of YZP in Treating AD.

## 3. Results

### 3.1. Screening for Active Compounds of YZP

**Figure 1:**
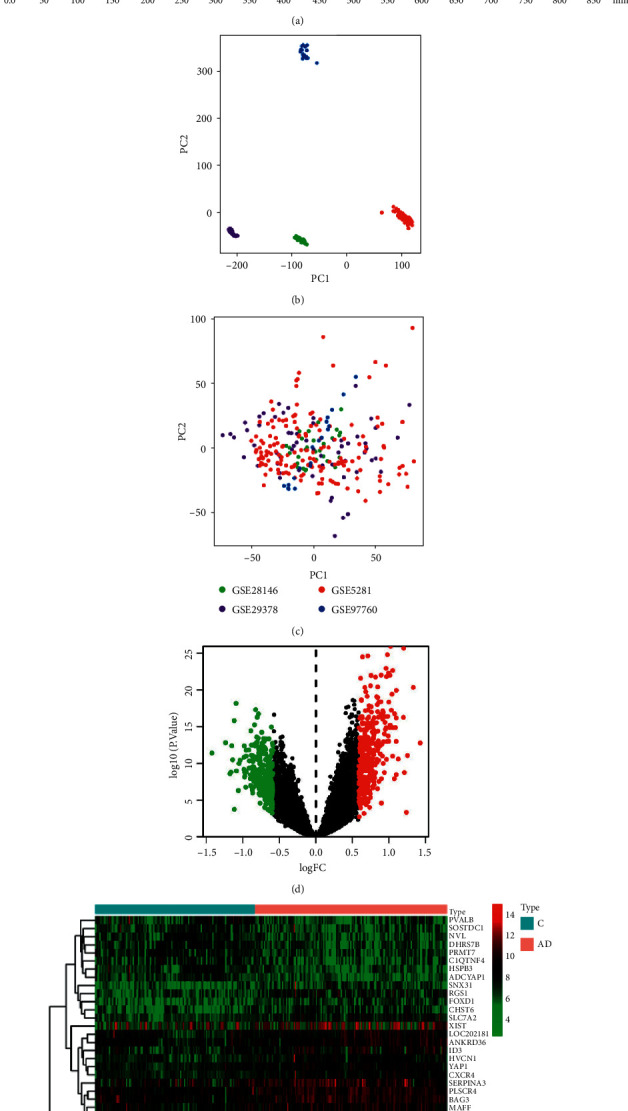
Fingerprint of Yuanzhi Powder (YZP) and AD differential genes (DEGs) screening. (a) Fingerprinting of YZP. Peak 5- 3,6′-disinapoylsucrose, peak 14- coptisine, peak 15- palmatine, peak 16- berberine. (b) PCA analysis before batch effect removal. (c) PCA analysis following batch effect removal. (d) Volcano map for DEGs. Red nodes represent upregulated genes, blue nodes represent downregulated genes, and black nodes represent no DEGs. (e) Heatmap of DEGs from AD and normal tissue. Red indicates higher gene expression, and blue indicates lower gene expression.

We compared the relative retention times of the peaks obtained from the HPLC method to detect YZP. Four peaks were analyzed (Figure 1(a)), including 3,6′-disinapoylsucrose (Peak 5), Coptisine (Peak 14), Palmatine (Peak 15), and Berberine (Peak 16). The Swiss Target Prediction database was imported, and target prediction was executed to obtain 282 targets for these four chemicals.

Besides TCMSP, HERB, and TCMID databases obtained 83 active compounds of YZP, and 224 targets were retrieved by UniProt rectification and reduplication. Consequently, by integrating the compounds from HPLC and public databases, 55 components of *Yuanzhi*, 22 components of *Renseng*, 15 components of *Fuling*, 14 components of *Huanglian*, and 4 components of *Shichangpu* were identified, as shown in Supplementary Table 1.

### 3.2. Screening for AD Differential Genes

We merged the data and eliminated the batch effect. The PCA diagram for the data set was obtained (Figures 1(b) and 1(c)). The four data sets were intersected together, and a batch of data was obtained for subsequent analysis. Using *P* < 0.05 and |log2FC| ＞ 0.584 as the screening criteria, the differential analysis was performed (Supplementary Table 2), and the differential volcano map was plotted (Figure 1(d)). The picture depicts 331 upregulated genes as red dots and 310 downregulated genes as green dots. We used the R software's *pheatmap* package to visualize the 50 upregulated and 50 downregulated genes with the most significant differential expression changes (Figure 1(e)).

### 3.3. Protein Interaction Network

**Figure 2:**
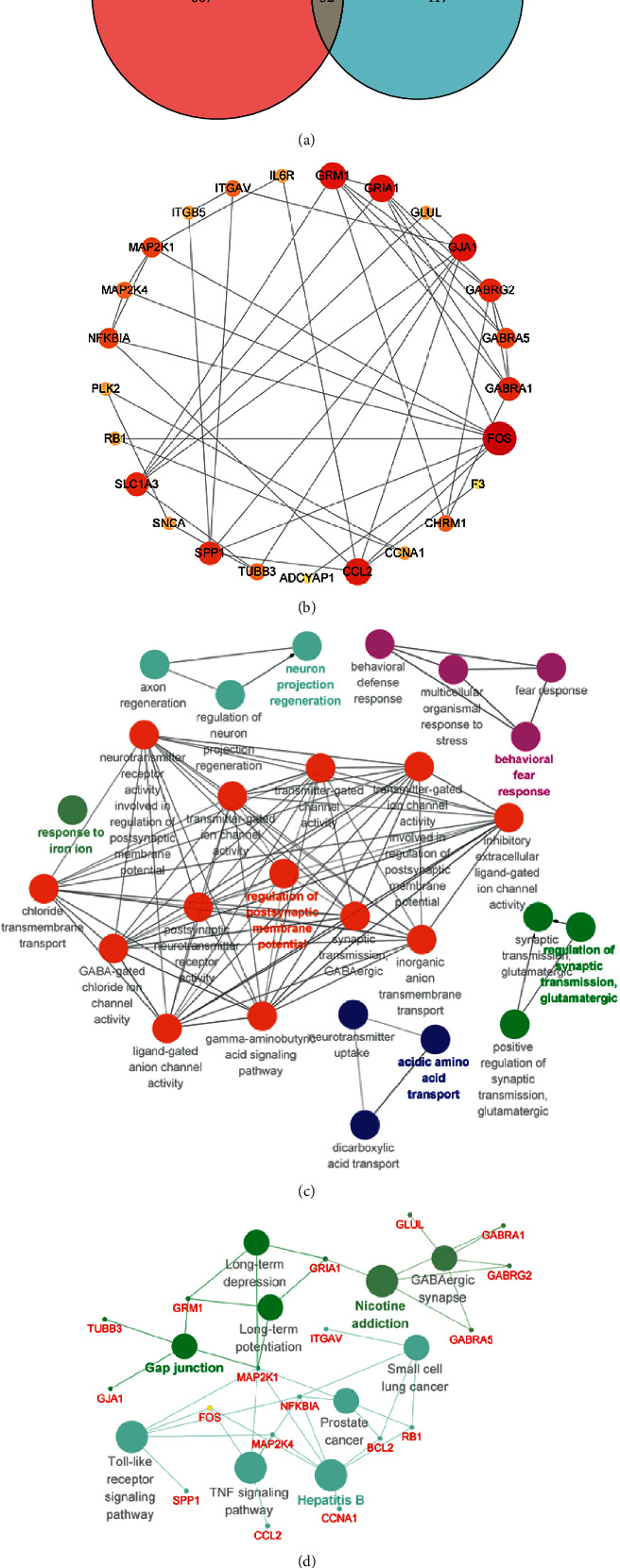
Screening and enrichment analysis of overlapping genes. (a) Venny map showing YZP-AD gene mapping. (b) PPI network exhibiting. (c) The enrichment of GO BP analyses of DEGs based on ClueGO enrichment analyses. (d) Enrichment of KEGG pathway via ClueGO enrichment analyses. ClueGO revealed correlations among channels by calculating the kappa coefficients.

32 intersection targets were obtained by taking the intersection of the active compounds of YZP and the AD differential genes (Figure 2(a)). We then imported the targets of YZP for AD into the STRING database and constructed a PPI network (Figure 2(b)). The darker the node's color is, the more interactions there are. Therefore, FOS, CCL2, GJA1, GRM1, and GRIA1 might be the primary target of YZP during AD treatment.

### 3.4. Components-Targets Network

**Figure 3:**
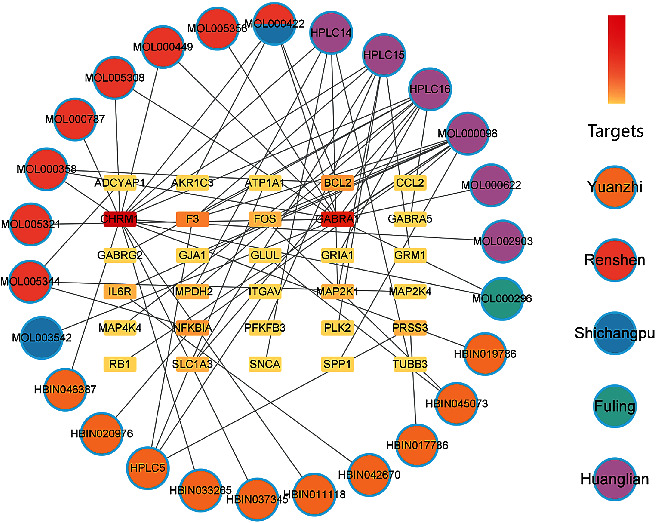
Active Components-Targets Network. The circle represents the composition of the herbal medicine, the rectangle represents the target point, and the redder color of the rectangle node indicates the number of degrees.

The 32 targets obtained above and the active components of YZP were used to form a components-targets network with 63 nodes and 140 edges (Figure 3). Circles depict the various chemical components of YZP, while the rectangles represent the predicted target. According to topological analysis, quercetin, berberine, 3,6′-disinapoylsucrose, coptisine, and palmatine have a higher degree value, which are the core positions in the network.

### 3.5. Enrichment Analysis

To uncover the potential mechanism of YZP in the treatment of AD, we used the Cytoscape plug-in ClueGO to perform an enrichment analysis of the GO biological process and KEGG pathway on 32 YZP for AD targets. Our results show that the GO biological process was enriched in AD-related YZP targets mainly involved in neuron projection regeneration, postsynaptic membrane potential, acidic amino acid transport, synaptic regulation of glutamatergic transmission, and behavioral fear response (Figure 2(c)). The results of KEGG enrichment show that ten different genes were enriched (Figure 2(d)), including gap junction, Toll-like receptor signaling pathway, TNF signaling pathway, and nicotine addiction. The gap junction proteins are associated with GJA1, TUBB3, and GRM1. Interestingly, the enrichment results primarily point to the nervous system.

### 3.6. The Result of Molecular Docking

Studies suggest that having a low affinity indicates a higher probability of its binding [18].

**Figure 4:**
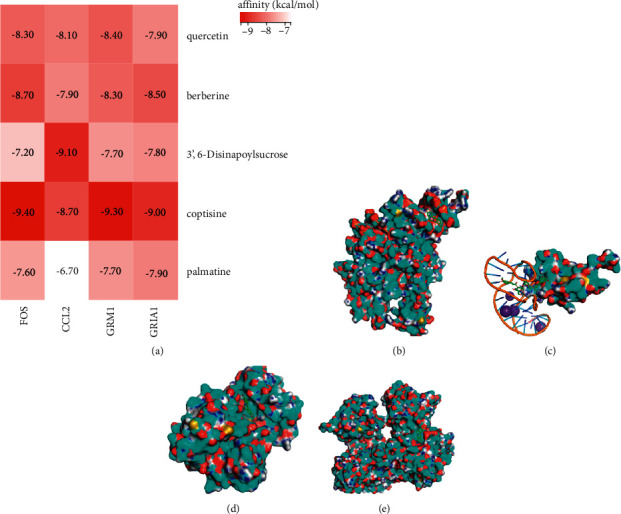
Molecular docking of crucial targets and ligands. (a) Heat map showing the results of molecular docking. (b) The conformations for FOS and coptisine. (c) The conformations for CCL2 and 3,6′-disinapoylsucrose. (d) The conformations for GRIA1 and coptisine. (e) The conformations for GRM1 and coptisine.

Following blind docking of ligands and proteins, the free energy values were utilized to generate a heat map using the R studio (Figure 4(a)). The binding affinity of FOS and coptisine was −9.4 kcal/mol (Figure 4(b)). CCL2 and 3,6′-disinapoylsucrose were bound with an affinity of −9.1 kcal/mol (Figure 4(c)). GRIA1 has a binding affinity of −9.3 kcal/mol for coptisine (Figure 4(d)). The binding affinity of GRM1 and coptisine was −9 kcal/mol (Figure 4(e)). The binding of FOS and coptisine had the lowest affinity of all the docking data, indicating the most critical binding affinity.

### 3.7. Immune Regulatory Network of YZP

**Figure 5:**
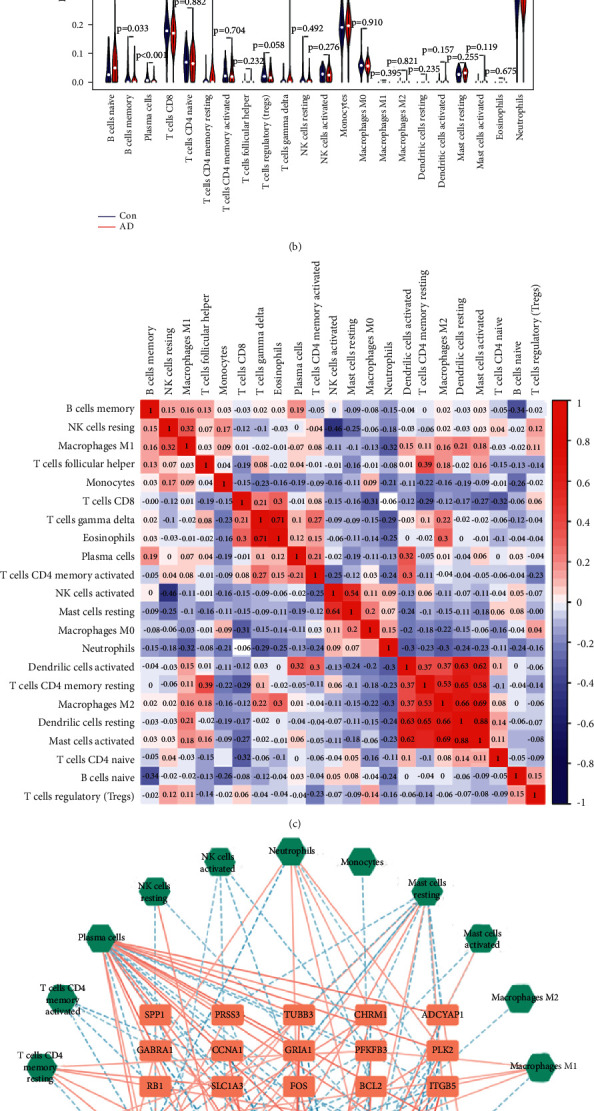
The immune infiltration between AD and controls and immune regulatory network. (a) The box plots show the relative percentage of different types of immune cells between AD patients and non-AD patients. (b) The difference in immune infiltration between AD (red) and controls (blue) (*P* values <0.05 indicated statistical significance). (c) The heat map shows the correlation in the infiltration of innate immune cells by CIBERSORT. (d) YZP-AD interaction plot of genes and immune-related molecules.

The CIBERSORT analysis indicated that the AD group had significantly higher naïve B cells and resting CD4 memory T cells but lower memory B cells and plasma cells (Figures 5(a) and 5(b)). The interaction of immune cells is shown in Figure 5(c). Our results show that the mast cell activation is significantly correlated with dendritic cell activation, resting CD4 memory T cells, macrophages M2, and resting dendritic cells.

We built an immune regulatory network to understand better the possible immunological mechanism of YZP in AD therapy (Figure 5(d)). The orange rectangle depicts YZP targets associated with AD, whereas the green diamond represents immune cells. The solid lines indicate a positive association between gene expression and immune cells, and the dashed lines indicate a negative correlation.

## 4. Discussion

**Figure 6:**
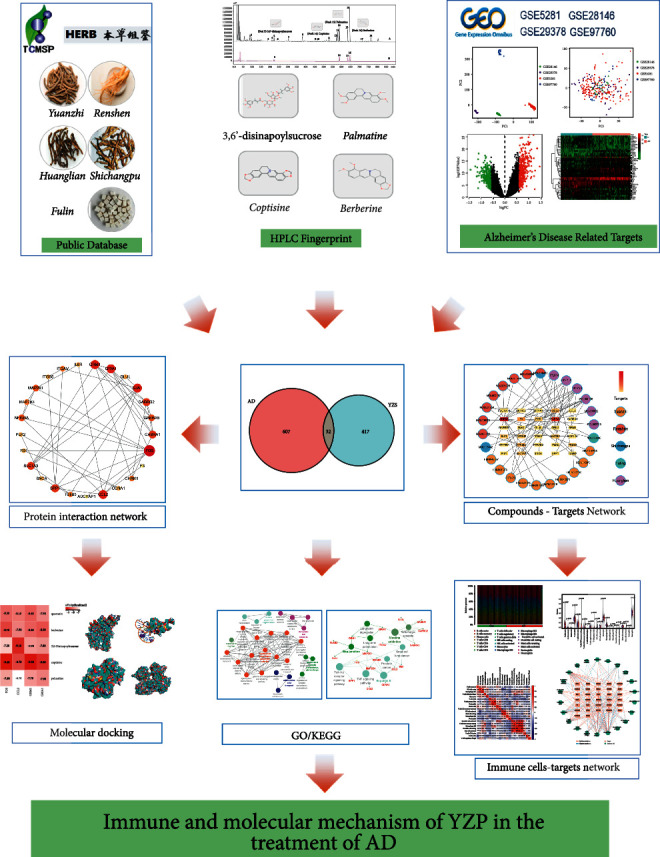
The flowchart of the study. The active compounds of YZP were conducted with HPLC fingerprint and various bibliographical databases and HPLC. Next, the genes associated with Alzheimer's disease were filtered by the GSE5281, GSE28146, GSE29378, and GSE97760. The active components-targets network and protein interaction network were then constructed by Cytoscape. The biological processes and signaling pathways were analyzed using the ClueGo Cytoscape plug-in. Molecular docking experiments were performed to verify the affinity of targets and ligands. Ultimately, the link between the hub genes and immune cell infiltration was assessed via CIBERSORT.

Traditional Chinese Medicine (TCM) prescription has the feature of being multi-component, multi-target, and multi-approach, and the therapeutic impact of TCM is not reflective of a single component. Therefore, regulating the quality of TCM decoctions and their derivatives is difficult. YZP, a TCM medicine, is extensively used to treat AD patients, but no quality control mechanism exists to ensure its effectiveness. HPLC has recently been extensively used to build a quality control technique for herbal medicine [19, 20]. However, it cannot discriminate between quality indicators, which indicate its effectiveness, and chemical markers. Network pharmacology involving HPLC has shown promise in illustrating TCM active compounds and pharmacological mechanisms [21]. Therefore, this study aims to explore the active compounds and immune mechanisms of YZP in AD treatment using HPLC and network pharmacology, The specific flow of this study is shown in Figure 6.

Network pharmacology results indicated that FOS, CCL2, and GJA1 were critical targets for YZP in treating AD. FOS can induce neuronal cell death during the onset of AD [22]. Similarly, FOS may stimulate phospholipid production and deplete phospholipids in the brain [23, 24]. CCL2, or C-C chemokine receptor 2, and increased levels of inflammation could enhance the chances of AD occurrence [25]. Connexin43 (Cx43), also known as GJA1, is an invertebrate connexin family member. GJA1 is a neuroprotective regulator of AD pathogenesis [26]. Using the WGCNA method, Zhang et al. discovered that GJA1 was a critical gene for the build-up of A*β* and phosphorylated tau, similar to our findings [27]. Our study found that core genes such as FOS, CCL2, and GJA1 represent promising pharmaceutical targets in AD.

By HPLC analysis, we obtained 18 peaks of YZP. We isolated four major components of YZP, including berberine, 3,6′-disinapoylsucrose, coptisine, and palmatine. Berberine can suppress BACE1 translation mediated by PERK/EIF2 signaling, decrease A*β* formation and cause neuronal cell death [28]. 3,6′-disinapoylsucrose, an active oligosaccharide ester component obtained from the roots of *Polygala tenuifolia*, can alleviate the pathological symptoms of AD through the activation of the cAMP/CREB/BDNF signaling pathway [29]. Coptisine effectively treated AD by inhibiting acetylcholinesterase [30], a standard treatment for the early stages of the most general form of AD. Besides, Coptisine may bind to multiple critical targets for YZP during AD treatment. Palmatine is a naturally occurring isoquinoline alkaloid that has been shown to have neuroprotective properties against amyloid-*β*-induced neurotoxicity [31]. Therefore, YZP can potentially treat AD with its multiple chemical components. Surprisingly, through the active component-target network of YZP, we found that quercetin is at the core of the network. In vitro and in vivo experiments have shown that quercetin plays a vital role in antioxidation, anticancer, and neuroprotection [32–34]. As one of the most common flavonoid compounds, qualitative and quantitative experiments on quercetin in YZP needs to be tested further.

The KEGG shows that gap junctions may be a critical pathway for YZP to treat AD. The gap junction is a unique membrane structure that connects two adjacent cells. It is essential in neurological diseases such as brain inflammation, epilepsy, neurodegenerative diseases, and ischemic stroke since it is a critical route of cell communication [35, 36]. Connexin (Cx), the fundamental unit of gap junctions, has been demonstrated in to be overexpressed in AD patients [37]. Cx can be expressed in various cells in the blood-brain barrier (BBB), and it can affect the permeability of BBB through mechanisms such as regulating Ca2+ signaling, releasing ATP, and directly triggering vascular endothelial cell death [38–40]. In the early stages of AD, abnormal function of BBB precedes pathogenic alterations and clinical symptoms. The gap junction channel aids ATP release into the extracellular area. Extracellular ATP may activate purinergic receptors and inflammasomes in many brain cells [41]. Inflammatory cytokines act on GJA1 in astrocytes, leading to neuroinflammation [42, 43]. As a result, YZP may effectively treat AD through gap junctions.

Immune infiltration is closely related to the clearance of A*β* in the brain [44]. Our research showed that the AD group had significantly higher naïve B cells and resting CD4 memory T cells, but lower memory B cells and plasma cells. Immature B cells first develop into naïve B cells in the spleen. B cells provide neuroprotection via nonspecific immunoglobulin and A*β*-specific antibodies [45]. Several studies have shown that CD4+ T cells are likely to have a role in the onset and progression of neuroinflammation and neurodegenerative disorders [46, 47]. We found that B cells and CD4 T cells may potentially be a promising new immunotherapy target for AD.

Moreover, we created an immune regulatory network of YZP in the treatment of AD by correlating the targets of YZP for AD with immune cells. We found that FOS was positively associated with naïve B cells and plasma cells. GJA1 also positively correlated with naïve B cells and negatively correlated with memory B cells. Also, GRM1 was negatively correlated with naïve B cells and positively correlated with plasma cells. However, there are very few relevant studies between AD and immune cells to confirm our findings, and further studies are needed to reveal the immunoregulatory mechanism of YZP on AD.

In this paper, we obtained the main active components of YZP by HPLC combined with public databases. HPLC has been widely used in various fields of qualitative and quantitative analysis because of its advantages such as high analysis speed, simple operation, high sensitivity, good reproducibility, and wide application range [48, 49]. However, HPLC has its limitations, for example, it cannot detect volatiles and provide accurate structures of compounds, and it is difficult to detect low levels of active components. These limitations make it difficult to determine all components in YZP, so we have supplemented the components using public databases to reduce the impact of HPLC limitations on the results. In future research, we will use more detection methods to perform a comprehensive analysis of YZP for different types of chemical components.

## 5. Conclusion

We investigated the possible molecular and immune mechanisms of YZP in AD therapy by using HPLC fingerprinting, network pharmacology, and gene chip technology. Our study identified quercetin, berberine, 3,6′-disinapoylsucrose, coptisine, and palmatine as the primary active ingredients of YZP. The gap junction was also found to be an essential signaling pathway in YZP for AD. Furthermore, we showed that the AD group had considerably higher naïve B cells and resting CD4 memory T cells but lower memory B cells and plasma cells. The ability to build an immune regulatory network was also established for the first time.

To summarize, our study highlights the potential of YZP in treating AD. YZP could act primarily due to the action of quercetin and berberine on the gap junctions. The immunotherapy effect of YZP on AD is mainly through GJA1 and GRM1 to regulate naïve B cells and plasma cells. These findings provide a direction for further exploration of the mechanism of YZP in the treatment of AD. However, these conclusions are all drawn based on theoretical simulations, so they must be verified through experiments.

## Figures and Tables

**Table 1:** Clinical information of GEO data sets.

GEO accession	Platforms	Normal	AD
GSE5281	GPL570	74	87
GSE28146	GPL570	8	22
GSE29378	GPL6947	32	31
GSE97760	GPL16699	10	9

## Data Availability

The original contributions presented in the study are included in the article/supplementary material. Further inquiries can be directed to the corresponding authors.

## Supplementary Materials

Supplementary Materials Supplementary Table 1: Active compounds of Yuanzhi powder. Supplementary Table 2: The differential genes of Alzheimer's disease.
