# Supplementary material for: Active Compounds and Targets of Yuanzhi Powder in Treating Alzheimer's Disease and Its Relationship with Immune Infiltration Based on HPLC Fingerprint and Network Pharmacology
Source: Evid Based Complement Alternat Med. 2022 Jul 15;2022:3389180. doi: 10.1155/2022/3389180 (PMC9307349; doi:10.1155/2022/3389180)
Supplement: Supplementary Materials — Supplementary Table 1: Active compounds of Yuanzhi powder. Supplementary Table 2: The differential genes of Alzheimer's disease. [file 3389180.f1.zip › 3389180.f1/Supplementary Table 1.docx]

**Supplementary Table 1：**Active compounds of YZS

| **Herbs** | **Mol ID** | **Molecule Name** | **OB/**  **%** | **DL** | **Sources** |
| --- | --- | --- | --- | --- | --- |
| Yuanzhi | HBIN001869 | 1,6-Dihydroxy-3,7-dimethoxyxanthone | 89.65 | 0.27 | HERB |
| Yuanzhi | HBIN002968 | 1-Peroxyferolide | 17.38 | 0.35 | HERB |
| Yuanzhi | HBIN011118 | 5,6,7-Trimethoxycoumarin | 32.54 | 0.12 | HERB |
| Yuanzhi | HBIN015652 | alpha-pinene | 46.25 | 0.05 | HERB |
| Yuanzhi | HBIN015681 | α-spinasterol | 42.98 | 0.76 | HERB |
| Yuanzhi | HBIN017786 | benzoic acid | 31.55 | 0.02 | HERB |
| Yuanzhi | HBIN019786 | carvacrol | 43.28 | 0.03 | HERB |
| Yuanzhi | HBIN020976 | citral | 22.52 | 0.02 | HERB |
| Yuanzhi | HBIN020993 | citronellal | 35.71 | 0.02 | HERB |
| Yuanzhi | HBIN028801 | Harman | 33.10 | 0.10 | HERB |
| Yuanzhi | HBIN028803 | harmine | 56.80 | 0.13 | HERB |
| Yuanzhi | HBIN029831 | hyperin | 6.94 | 0.77 | HERB |
| Yuanzhi | HBIN031102 | isoquercitrin | 1.86 | 0.77 | HERB |
| Yuanzhi | HBIN032760 | laurinaldehyde | 21.52 | 0.03 | HERB |
| Yuanzhi | HBIN032827 | leaf alcohol | 62.74 | 0.01 | HERB |
| Yuanzhi | HBIN033245 | limonene | 38.09 | 0.02 | HERB |
| Yuanzhi | HBIN033265 | linalool | 39.80 | 0.02 | HERB |
| Yuanzhi | HBIN037343 | Norharman | 18.88 | 0.08 | HERB |
| Yuanzhi | HBIN037345 | Norhyoscyamine | 60.34 | 0.17 | HERB |
| Yuanzhi | HBIN037700 | ocimene | 21.43 | 0.02 | HERB |
| Yuanzhi | HBIN038158 | Onjisaponin F | 7.70 | 0.01 | TCM-ID |
| Yuanzhi | HBIN038159 | Onjisaponin G | 7.89 | 0.01 | TCM-ID |
| Yuanzhi | HBIN039299 | Perlolyrine | 65.95 | 0.27 | HERB |
| Yuanzhi | HBIN041726 | quercitrin | 4.04 | 0.74 | HERB |
| Yuanzhi | HBIN042670 | rutin | 3.20 | 0.68 | HERB |
| Yuanzhi | HBIN045073 | sucrose | 7.17 | 0.23 | HERB |
| Yuanzhi | HBIN046387 | thymol | 41.47 | 0.03 | HERB |
| Yuanzhi | HPLC5 | 3',6-Disinapoylsucrose | - | - | HPLC |
| Fuling | MOL000273 | (2R)-2-[(3S,5R,10S,13R,14R,16R,17R)-3,16-dihydroxy-4,4,10,13,14-pentamethyl-2,3,5,6,12,15,16,17-octahydro-1H-cyclopenta[a]phenanthren-17-yl]-6-methylhept-5-enoic acid | 30.93 | 0.81 | TCMSP |
| Fuling | MOL000275 | trametenolic acid | 38.71 | 0.80 | TCMSP |
| Fuling | MOL000276 | 7,9(11)-dehydropachymic acid | 35.11 | 0.81 | TCMSP |
| Fuling | MOL000279 | Cerevisterol | 37.96 | 0.77 | TCMSP |
| Fuling | MOL000280 | (2R)-2-[(3S,5R,10S,13R,14R,16R,17R)-3,16-dihydroxy-4,4,10,13,14-pentamethyl-2,3,5,6,12,15,16,17-octahydro-1H-cyclopenta[a]phenanthren-17-yl]-5-isopropyl-hex-5-enoic acid | 31.07 | 0.82 | TCMSP |
| Fuling | MOL000282 | ergosta-7,22E-dien-3beta-ol | 43.51 | 0.72 | TCMSP |
| Fuling | MOL000283 | Ergosterol peroxide | 40.36 | 0.81 | TCMSP |
| Fuling | MOL000285 | (2R)-2-[(5R,10S,13R,14R,16R,17R)-16-hydroxy-3-keto-4,4,10,13,14-pentamethyl-1,2,5,6,12,15,16,17-octahydrocyclopenta[a]phenanthren-17-yl]-5-isopropyl-hex-5-enoic acid | 38.26 | 0.82 | TCMSP |
| Fuling | MOL000287 | 3beta-Hydroxy-24-methylene-8-lanostene-21-oic acid | 38.70 | 0.81 | TCMSP |
| Fuling | MOL000289 | pachymic acid | 33.63 | 0.81 | TCMSP |
| Fuling | MOL000290 | Poricoic acid A | 30.61 | 0.76 | TCMSP |
| Fuling | MOL000291 | Poricoic acid B | 30.52 | 0.75 | TCMSP |
| Fuling | MOL000292 | poricoic acid C | 38.15 | 0.75 | TCMSP |
| Fuling | MOL000296 | hederagenin | 36.91 | 0.75 | TCMSP |
| Fuling | MOL000300 | dehydroeburicoic acid | 44.17 | 0.83 | TCMSP |
| huanglian | MOL001454 | berberine | 36.86 | 0.78 | TCMSP |
| Huanglian | MOL013352 | Obacunone | 43.29 | 0.77 | TCMSP |
| Huanglian | MOL002894 | berberrubine | 35.74 | 0.73 | TCMSP |
| Huanglian | MOL002897 | epiberberine | 43.09 | 0.78 | TCMSP |
| Huanglian | MOL002903 | (R)-Canadine | 55.37 | 0.77 | TCMSP |
| Huanglian | MOL002904 | Berlambine | 36.68 | 0.82 | TCMSP |
| Huanglian | MOL002907 | Corchoroside A_qt | 104.95 | 0.78 | TCMSP |
| Huanglian | MOL000622 | Magnograndiolide | 63.71 | 0.19 | TCMSP |
| Huanglian | MOL000762 | Palmidin A | 35.36 | 0.65 | TCMSP |
| Huanglian | MOL000785 | palmatine | 64.60 | 0.65 | TCMSP |
| Huanglian | MOL000098 | quercetin | 46.43 | 0.28 | TCMSP |
| Huanglian | MOL001458 | coptisine | 30.67 | 0.86 | TCMSP |
| Huanglian | MOL002668 | Worenine | 45.83 | 0.87 | TCMSP |
| Huanglian | MOL008647 | Moupinamide | 86.71 | 0.26 | TCMSP |
| Huanglian | HPLC14 | Coptisine | - | - | HPLC |
| Huanglian | HPLC15 | Palmatine | - | - | HPLC |
| Huanglian | HPLC16 | Berberine | - | - | HPLC |
| Renshen | MOL002879 | Diop | 43.59 | 0.39 | TCMSP |
| Renshen | MOL000449 | Stigmasterol | 43.83 | 0.76 | TCMSP |
| Renshen | MOL000358 | beta-sitosterol | 36.91 | 0.75 | TCMSP |
| Renshen | MOL003648 | Inermin | 65.83 | 0.54 | TCMSP |
| Renshen | MOL000422 | kaempferol | 41.88 | 0.24 | TCMSP |
| Renshen | MOL004492 | Chrysanthemaxanthin | 38.72 | 0.58 | TCMSP |
| Renshen | MOL005308 | Aposiopolamine | 66.65 | 0.22 | TCMSP |
| Renshen | MOL005314 | Celabenzine | 101.88 | 0.49 | TCMSP |
| Renshen | MOL005317 | Deoxyharringtonine | 39.27 | 0.81 | TCMSP |
| Renshen | MOL005318 | Dianthramine | 40.45 | 0.20 | TCMSP |
| Renshen | MOL005320 | arachidonate | 45.57 | 0.20 | TCMSP |
| Renshen | MOL005321 | Frutinone A | 65.90 | 0.34 | TCMSP |
| Renshen | MOL005344 | ginsenoside rh2 | 36.32 | 0.56 | TCMSP |
| Renshen | MOL005348 | Ginsenoside-Rh4_qt | 31.11 | 0.78 | TCMSP |
| Renshen | MOL005356 | Girinimbin | 61.22 | 0.31 | TCMSP |
| Renshen | MOL005357 | Gomisin B | 31.99 | 0.83 | TCMSP |
| Renshen | MOL005360 | malkangunin | 57.71 | 0.63 | TCMSP |
| Renshen | MOL005376 | Panaxadiol | 33.09 | 0.79 | TCMSP |
| Renshen | MOL005384 | suchilactone | 57.52 | 0.56 | TCMSP |
| Renshen | MOL005399 | alexandrin_qt | 36.91 | 0.75 | TCMSP |
| Renshen | MOL005401 | ginsenoside Rg5_qt | 39.56 | 0.79 | TCMSP |
| Renshen | MOL000787 | Fumarine | 59.26 | 0.83 | TCMSP |
| Shichangpu | MOL003542 | 8-Isopentenyl-kaempferol | 38.04 | 0.39 | TCMSP |
| Shichangpu | MOL003576 | (1R,3aS,4R,6aS)-1,4-bis(3,4-dimethoxyphenyl)-1,3,3a,4,6,6a-hexahydrofuro[4,3-c]furan | 52.35 | 0.62 | TCMSP |
| Shichangpu | MOL003578 | Cycloartenol | 38.69 | 0.78 | TCMSP |
| Shichangpu | MOL000422 | kaempferol | 41.88 | 0.24 | TCMSP |
