# Supplementary material for: Active Compounds and Targets of Yuanzhi Powder in Treating Alzheimer's Disease and Its Relationship with Immune Infiltration Based on HPLC Fingerprint and Network Pharmacology
Source: Evid Based Complement Alternat Med. 2022 Jul 15;2022:3389180. doi: 10.1155/2022/3389180 (PMC9307349; doi:10.1155/2022/3389180)
Supplement: Supplementary Materials — Supplementary Table 1: Active compounds of Yuanzhi powder. Supplementary Table 2: The differential genes of Alzheimer's disease. [file 3389180.f1.zip › 3389180.f1/Supplementary Table 2.docx]

**Supplementary Table 2：**The differential genes of Alzheimer's Disease

| id | logFC |  | AveExpr | t | P.Value | adj.P.Val | B |
| --- | --- | --- | --- | --- | --- | --- | --- |
| PFKFB3 | 1.022573 |  | 11.55557 | 11.90017 | 1.19E-26 | 1.56E-22 | 49.71045 |
| ZFP36L1 | 1.201728 |  | 8.869689 | 11.82854 | 2.09E-26 | 1.56E-22 | 49.15593 |
| ERBB2IP | 0.979513 |  | 8.873355 | 11.56884 | 1.62E-25 | 8.04E-22 | 47.15464 |
| NAV2 | 0.710542 |  | 8.14006 | 11.52124 | 2.36E-25 | 8.77E-22 | 46.78943 |
| PPFIA1 | 0.637262 |  | 8.538232 | 11.48192 | 3.21E-25 | 9.55E-22 | 46.48808 |
| BCL6 | 0.960023 |  | 9.967455 | 11.02077 | 1.16E-23 | 2.88E-20 | 42.98178 |
| ITPKB | 1.050384 |  | 8.477299 | 10.93482 | 2.25E-23 | 4.79E-20 | 42.33417 |
| MALAT1 | 0.994665 |  | 11.87378 | 10.83483 | 4.85E-23 | 9.03E-20 | 41.5833 |
| SASH1 | 0.892967 |  | 9.79955 | 10.7447 | 9.68E-23 | 1.45E-19 | 40.90878 |
| DTNA | 1.011213 |  | 10.16001 | 10.74129 | 9.94E-23 | 1.45E-19 | 40.88331 |
| TNPO1 | 0.756314 |  | 10.24177 | 10.73184 | 1.07E-22 | 1.45E-19 | 40.81269 |
| TGFBR3 | 0.965289 |  | 9.216503 | 10.68475 | 1.53E-22 | 1.90E-19 | 40.46135 |
| AFF1 | 0.817088 |  | 8.388327 | 10.6652 | 1.78E-22 | 2.04E-19 | 40.31566 |
| STAG2 | 0.612404 |  | 9.072693 | 10.6211 | 2.49E-22 | 2.64E-19 | 39.98742 |
| ADD3 | 0.748003 |  | 11.08365 | 10.30584 | 2.70E-21 | 2.68E-18 | 37.65763 |
| LPP | 0.846447 |  | 8.765715 | 10.25843 | 3.86E-21 | 3.59E-18 | 37.30987 |
| GFAP | 1.332672 |  | 11.43469 | 10.23615 | 4.56E-21 | 3.84E-18 | 37.14666 |
| RHOQ | 0.670009 |  | 9.856147 | 10.23376 | 4.64E-21 | 3.84E-18 | 37.12921 |
| CXCR4 | 1.10012 |  | 7.650239 | 10.11263 | 1.15E-20 | 8.99E-18 | 36.24491 |
| BBX | 0.681214 |  | 9.412481 | 10.05702 | 1.74E-20 | 1.29E-17 | 35.84048 |
| YAP1 | 1.041962 |  | 7.88742 | 9.953715 | 3.74E-20 | 2.65E-17 | 35.09198 |
| UNG | 0.702555 |  | 8.752937 | 9.86138 | 7.40E-20 | 5.01E-17 | 34.42599 |
| QKI | 0.832417 |  | 9.652074 | 9.846907 | 8.23E-20 | 5.33E-17 | 34.32186 |
| NFKBIA | 0.846075 |  | 9.888252 | 9.811344 | 1.07E-19 | 6.63E-17 | 34.06632 |
| SORBS1 | 0.622082 |  | 9.231353 | 9.717941 | 2.12E-19 | 1.26E-16 | 33.39724 |
| EPC1 | 0.623326 |  | 8.685299 | 9.683112 | 2.74E-19 | 1.51E-16 | 33.14854 |
| LIFR | 0.889401 |  | 8.361924 | 9.629175 | 4.06E-19 | 2.08E-16 | 32.76425 |
| NSUN6 | 0.998151 |  | 8.53437 | 9.615801 | 4.47E-19 | 2.22E-16 | 32.66912 |
| RFX4 | 0.861095 |  | 7.254871 | 9.607385 | 4.76E-19 | 2.28E-16 | 32.6093 |
| ATP6V1G2 | -1.09133 |  | 9.962962 | -9.56231 | 6.60E-19 | 3.07E-16 | 32.28932 |
| METTL7A | 0.840479 |  | 10.15187 | 9.502453 | 1.02E-18 | 4.60E-16 | 31.86556 |
| PTMA | 0.729052 |  | 11.0052 | 9.459272 | 1.39E-18 | 5.93E-16 | 31.56068 |
| PALLD | 0.758309 |  | 8.938189 | 9.415777 | 1.91E-18 | 7.89E-16 | 31.25429 |
| IDH3G | -0.82316 |  | 8.405591 | -9.28963 | 4.72E-18 | 1.76E-15 | 30.36977 |
| ITGB8 | 0.711526 |  | 7.1996 | 9.274069 | 5.28E-18 | 1.92E-15 | 30.26107 |
| PLSCR4 | 1.032697 |  | 9.144554 | 9.263028 | 5.71E-18 | 2.03E-15 | 30.18401 |
| ANKRD12 | 0.677203 |  | 9.679069 | 9.242532 | 6.62E-18 | 2.29E-15 | 30.04109 |
| GRAMD3 | 0.914849 |  | 9.168185 | 9.23411 | 7.03E-18 | 2.38E-15 | 29.98241 |
| ANP32B | 0.843991 |  | 11.58142 | 9.198858 | 9.03E-18 | 2.99E-15 | 29.73709 |
| SOX2 | 0.761188 |  | 8.952616 | 9.182243 | 1.02E-17 | 3.29E-15 | 29.62164 |
| AHNAK | 0.920787 |  | 8.449177 | 9.16751 | 1.13E-17 | 3.58E-15 | 29.51936 |
| GJA1 | 1.075122 |  | 11.89362 | 9.108739 | 1.71E-17 | 5.10E-15 | 29.11221 |
| ATP5B | -0.78376 |  | 11.24508 | -9.10003 | 1.82E-17 | 5.32E-15 | 29.05202 |
| ID4 | 0.892881 |  | 8.624435 | 9.096651 | 1.87E-17 | 5.35E-15 | 29.02863 |
| TOB1 | 0.908044 |  | 9.774791 | 9.017891 | 3.26E-17 | 8.82E-15 | 28.48559 |
| MED13L | 0.626359 |  | 8.549384 | 8.991129 | 3.94E-17 | 1.00E-14 | 28.30164 |
| SRGN | 0.982569 |  | 9.32017 | 8.989814 | 3.97E-17 | 1.00E-14 | 28.2926 |
| CPEB4 | 0.629817 |  | 9.507949 | 8.983187 | 4.16E-17 | 1.02E-14 | 28.24711 |
| ACACB | 0.963817 |  | 9.149031 | 8.982949 | 4.17E-17 | 1.02E-14 | 28.24547 |
| ESF1 | 0.724458 |  | 8.393166 | 8.957839 | 4.98E-17 | 1.18E-14 | 28.07323 |
| CMBL | 0.855963 |  | 9.803024 | 8.952154 | 5.18E-17 | 1.18E-14 | 28.03427 |
| ACOT7 | -0.80116 |  | 8.906701 | -8.95108 | 5.22E-17 | 1.18E-14 | 28.0269 |
| SLC7A2 | 1.198003 |  | 6.935083 | 8.941038 | 5.60E-17 | 1.24E-14 | 27.95814 |
| KLHL24 | 0.604613 |  | 9.244631 | 8.937414 | 5.74E-17 | 1.26E-14 | 27.93333 |
| PLOD2 | 0.797829 |  | 8.429888 | 8.927176 | 6.17E-17 | 1.33E-14 | 27.86326 |
| ANKRD36 | 1.099115 |  | 8.482662 | 8.916968 | 6.63E-17 | 1.40E-14 | 27.79345 |
| PTCH1 | 0.63209 |  | 7.871893 | 8.916418 | 6.66E-17 | 1.40E-14 | 27.78969 |
| CTDSP2 | 0.598823 |  | 9.887975 | 8.90115 | 7.41E-17 | 1.53E-14 | 27.68535 |
| CHSY1 | 0.705159 |  | 10.37604 | 8.882994 | 8.41E-17 | 1.69E-14 | 27.5614 |
| HIPK2 | 0.914604 |  | 10.05998 | 8.866717 | 9.43E-17 | 1.87E-14 | 27.45041 |
| INA | -1.11463 |  | 10.1343 | -8.79701 | 1.53E-16 | 2.93E-14 | 26.97631 |
| RHOBTB3 | 0.776636 |  | 9.369772 | 8.786066 | 1.65E-16 | 3.12E-14 | 26.90208 |
| PMP2 | 0.945252 |  | 10.74247 | 8.717761 | 2.66E-16 | 4.95E-14 | 26.43988 |
| ATP8B1 | 0.829354 |  | 8.277658 | 8.706741 | 2.87E-16 | 5.21E-14 | 26.3655 |
| NUCKS1 | 0.634868 |  | 11.38609 | 8.695534 | 3.10E-16 | 5.56E-14 | 26.28991 |
| TOB2 | 0.713973 |  | 8.515786 | 8.687444 | 3.28E-16 | 5.81E-14 | 26.23538 |
| PSAT1 | 0.807201 |  | 9.763062 | 8.68573 | 3.32E-16 | 5.81E-14 | 26.22383 |
| ZFP36L2 | 0.756723 |  | 8.09633 | 8.677616 | 3.51E-16 | 6.08E-14 | 26.16917 |
| KANK1 | 0.661196 |  | 8.331087 | 8.669663 | 3.71E-16 | 6.34E-14 | 26.11562 |
| VCAN | 0.763054 |  | 9.187379 | 8.657248 | 4.04E-16 | 6.83E-14 | 26.03209 |
| BCL2 | 0.637722 |  | 8.035727 | 8.648252 | 4.30E-16 | 7.19E-14 | 25.97161 |
| MLLT11 | -0.83819 |  | 12.20611 | -8.60942 | 5.62E-16 | 9.29E-14 | 25.71091 |
| GRAMD1C | 1.015441 |  | 8.320469 | 8.595215 | 6.19E-16 | 1.00E-13 | 25.61575 |
| PTTG1IP | 0.699574 |  | 10.79929 | 8.537461 | 9.21E-16 | 1.46E-13 | 25.22966 |
| RAP1GDS1 | -0.60748 |  | 8.991314 | -8.50795 | 1.13E-15 | 1.77E-13 | 25.03299 |
| NLN | 0.724552 |  | 8.823117 | 8.50207 | 1.17E-15 | 1.82E-13 | 24.99381 |
| SLC35E1 | 0.778193 |  | 10.03493 | 8.500238 | 1.19E-15 | 1.82E-13 | 24.98162 |
| MT1M | 1.008796 |  | 8.775848 | 8.481321 | 1.35E-15 | 2.04E-13 | 24.8558 |
| BAG3 | 1.088196 |  | 9.20631 | 8.480893 | 1.36E-15 | 2.04E-13 | 24.85296 |
| HVCN1 | 1.051564 |  | 7.470446 | 8.475453 | 1.41E-15 | 2.09E-13 | 24.81681 |
| CREBBP | 0.587529 |  | 8.781371 | 8.474091 | 1.42E-15 | 2.09E-13 | 24.80776 |
| RBM25 | 0.705738 |  | 10.48445 | 8.470996 | 1.45E-15 | 2.12E-13 | 24.7872 |
| SRRM2 | 0.961705 |  | 10.85874 | 8.468347 | 1.48E-15 | 2.13E-13 | 24.7696 |
| DDX59 | 0.720909 |  | 8.796337 | 8.46658 | 1.49E-15 | 2.14E-13 | 24.75787 |
| SEPP1 | 0.950722 |  | 9.456631 | 8.401279 | 2.33E-15 | 3.24E-13 | 24.32525 |
| KIF5B | 0.697656 |  | 9.770119 | 8.389485 | 2.52E-15 | 3.48E-13 | 24.24733 |
| KTN1 | 0.615503 |  | 10.12699 | 8.384428 | 2.61E-15 | 3.55E-13 | 24.21394 |
| FOXO1 | 0.661729 |  | 8.21199 | 8.383682 | 2.63E-15 | 3.55E-13 | 24.20902 |
| NOTCH2 | 0.79692 |  | 8.5553 | 8.349663 | 3.31E-15 | 4.40E-13 | 23.9847 |
| FHL2 | -0.88863 |  | 8.134717 | -8.33136 | 3.74E-15 | 4.93E-13 | 23.86423 |
| TMEM123 | 0.848325 |  | 9.527346 | 8.31997 | 4.04E-15 | 5.27E-13 | 23.78935 |
| PON2 | 0.827804 |  | 10.27515 | 8.303132 | 4.53E-15 | 5.81E-13 | 23.67875 |
| NFIA | 0.771074 |  | 9.548679 | 8.282235 | 5.21E-15 | 6.63E-13 | 23.54168 |
| NEDD1 | 0.670506 |  | 7.13747 | 8.26696 | 5.77E-15 | 7.28E-13 | 23.44162 |
| MPP1 | -0.73991 |  | 8.845309 | -8.2642 | 5.88E-15 | 7.34E-13 | 23.42358 |
| EMX2 | 0.9379 |  | 8.646969 | 8.19444 | 9.39E-15 | 1.14E-12 | 22.96807 |
| MEGF10 | 0.793714 |  | 9.104708 | 8.184134 | 1.01E-14 | 1.21E-12 | 22.90098 |
| DNAJC1 | 0.669666 |  | 8.8826 | 8.17796 | 1.05E-14 | 1.24E-12 | 22.86081 |
| RIN2 | 0.795777 |  | 8.895427 | 8.141017 | 1.34E-14 | 1.54E-12 | 22.62084 |
| SEMA4C | 0.670071 |  | 9.500701 | 8.102487 | 1.73E-14 | 1.96E-12 | 22.37127 |
| ZIC2 | 0.988891 |  | 8.936002 | 8.095193 | 1.82E-14 | 2.04E-12 | 22.3241 |
| IFRD1 | 0.684662 |  | 9.032123 | 8.061067 | 2.28E-14 | 2.54E-12 | 22.10378 |
| GPI | -0.80851 |  | 9.95932 | -8.0431 | 2.57E-14 | 2.81E-12 | 21.98801 |
| FAM84B | 0.711067 |  | 7.658203 | 8.026254 | 2.87E-14 | 3.12E-12 | 21.87961 |
| GLIS3 | 0.785479 |  | 7.154805 | 8.014832 | 3.10E-14 | 3.30E-12 | 21.80619 |
| TPD52L1 | 0.691664 |  | 9.174176 | 8.0091 | 3.22E-14 | 3.40E-12 | 21.76937 |
| GAS2L1 | 0.621227 |  | 9.173269 | 8.002276 | 3.37E-14 | 3.53E-12 | 21.72556 |
| CCNG1 | 0.595156 |  | 8.944891 | 8.000536 | 3.41E-14 | 3.55E-12 | 21.71439 |
| ATRNL1 | -0.78722 |  | 7.869454 | -7.98984 | 3.65E-14 | 3.74E-12 | 21.64578 |
| EMP1 | 0.712984 |  | 7.181347 | 7.989132 | 3.67E-14 | 3.74E-12 | 21.64123 |
| PHF6 | 0.698918 |  | 8.317441 | 7.978583 | 3.94E-14 | 3.97E-12 | 21.57361 |
| CRYM | -0.71045 |  | 9.409689 | -7.97802 | 3.95E-14 | 3.97E-12 | 21.57003 |
| ANLN | 0.78981 |  | 8.485513 | 7.97667 | 3.99E-14 | 3.98E-12 | 21.56136 |
| BMPR1B | 0.714059 |  | 7.709787 | 7.961848 | 4.39E-14 | 4.33E-12 | 21.46646 |
| SLC25A4 | -0.64118 |  | 9.046019 | -7.9584 | 4.50E-14 | 4.37E-12 | 21.44441 |
| PSMB3 | -0.68287 |  | 9.665557 | -7.94665 | 4.86E-14 | 4.63E-12 | 21.36925 |
| UACA | 0.621331 |  | 7.320051 | 7.939278 | 5.10E-14 | 4.83E-12 | 21.32218 |
| CD44 | 0.640841 |  | 7.425145 | 7.928768 | 5.46E-14 | 5.15E-12 | 21.25507 |
| ABCA1 | 0.718497 |  | 8.153234 | 7.885074 | 7.27E-14 | 6.76E-12 | 20.97669 |
| CEBPB | 0.768287 |  | 10.55396 | 7.874903 | 7.77E-14 | 7.14E-12 | 20.91203 |
| KLF15 | 0.855073 |  | 8.114911 | 7.858063 | 8.67E-14 | 7.92E-12 | 20.80509 |
| DDIT4 | 0.997038 |  | 10.2992 | 7.841441 | 9.67E-14 | 8.70E-12 | 20.69967 |
| FGF12 | -0.70761 |  | 7.476637 | -7.84087 | 9.70E-14 | 8.70E-12 | 20.69607 |
| MAFF | 1.098503 |  | 8.499273 | 7.836714 | 9.97E-14 | 8.89E-12 | 20.66971 |
| TBL1X | 0.702624 |  | 8.337131 | 7.830572 | 1.04E-13 | 9.18E-12 | 20.63081 |
| GEM | 0.971086 |  | 6.881464 | 7.829958 | 1.04E-13 | 9.18E-12 | 20.62693 |
| WWTR1 | 0.751362 |  | 6.97853 | 7.822017 | 1.10E-13 | 9.60E-12 | 20.57666 |
| LRP4 | 0.925529 |  | 9.459581 | 7.82129 | 1.10E-13 | 9.60E-12 | 20.57206 |
| HSD17B7 | 0.655804 |  | 9.865775 | 7.80486 | 1.23E-13 | 1.04E-11 | 20.46816 |
| ZFHX3 | 0.613963 |  | 7.370544 | 7.800015 | 1.27E-13 | 1.05E-11 | 20.43755 |
| ATP6V1E1 | -0.80063 |  | 11.22587 | -7.79908 | 1.27E-13 | 1.05E-11 | 20.43162 |
| NRXN3 | -0.81164 |  | 8.37411 | -7.77758 | 1.46E-13 | 1.17E-11 | 20.29599 |
| CHGB | -1.23484 |  | 9.325276 | -7.77217 | 1.52E-13 | 1.19E-11 | 20.26184 |
| RTN3 | -0.73041 |  | 10.97997 | -7.76319 | 1.61E-13 | 1.25E-11 | 20.20527 |
| RGS1 | 1.428429 |  | 6.802574 | 7.757633 | 1.67E-13 | 1.29E-11 | 20.1703 |
| ZNF160 | 0.826854 |  | 9.41906 | 7.750503 | 1.75E-13 | 1.34E-11 | 20.12543 |
| ATP1A2 | 0.829123 |  | 11.27977 | 7.749822 | 1.75E-13 | 1.34E-11 | 20.12115 |
| PSMB7 | -0.59187 |  | 9.524353 | -7.74253 | 1.84E-13 | 1.40E-11 | 20.07529 |
| VASN | 0.77348 |  | 7.330001 | 7.734691 | 1.93E-13 | 1.44E-11 | 20.02602 |
| OSMR | 0.695494 |  | 6.636136 | 7.730904 | 1.98E-13 | 1.47E-11 | 20.00223 |
| SLC38A2 | 0.755413 |  | 11.04894 | 7.729831 | 1.99E-13 | 1.47E-11 | 19.99549 |
| WDR82 | 0.599956 |  | 11.25505 | 7.70007 | 2.42E-13 | 1.75E-11 | 19.80881 |
| FAM107B | 0.903326 |  | 9.880442 | 7.697846 | 2.45E-13 | 1.76E-11 | 19.79488 |
| NOTCH2NL | 0.877777 |  | 10.0807 | 7.693857 | 2.52E-13 | 1.80E-11 | 19.7699 |
| REEP1 | -0.83796 |  | 9.948334 | -7.68945 | 2.59E-13 | 1.83E-11 | 19.74233 |
| REEP3 | 0.700326 |  | 7.4431 | 7.670882 | 2.92E-13 | 2.05E-11 | 19.62617 |
| IL6R | 0.628202 |  | 6.900656 | 7.669109 | 2.95E-13 | 2.06E-11 | 19.61509 |
| AQP4 | 0.838638 |  | 9.839347 | 7.668038 | 2.97E-13 | 2.07E-11 | 19.6084 |
| NCALD | -0.8519 |  | 9.055924 | -7.66577 | 3.01E-13 | 2.08E-11 | 19.5942 |
| DYNC1I1 | -0.87912 |  | 9.359995 | -7.66116 | 3.10E-13 | 2.13E-11 | 19.56543 |
| CA10 | -0.82608 |  | 8.134534 | -7.65127 | 3.31E-13 | 2.25E-11 | 19.50368 |
| DHCR24 | -0.8653 |  | 8.771545 | -7.65029 | 3.33E-13 | 2.25E-11 | 19.49762 |
| DPP6 | -0.58701 |  | 9.671565 | -7.64103 | 3.53E-13 | 2.36E-11 | 19.43985 |
| SOX9 | 0.827755 |  | 9.546291 | 7.622364 | 3.98E-13 | 2.62E-11 | 19.32358 |
| CHRM1 | -1.14961 |  | 7.7767 | -7.62098 | 4.02E-13 | 2.63E-11 | 19.31497 |
| NME1 | -0.80764 |  | 9.49295 | -7.62013 | 4.04E-13 | 2.64E-11 | 19.30964 |
| TJP1 | 0.631518 |  | 10.0508 | 7.61666 | 4.13E-13 | 2.68E-11 | 19.28808 |
| MT2A | 0.876897 |  | 12.18341 | 7.615601 | 4.16E-13 | 2.68E-11 | 19.28149 |
| SVOP | -0.72117 |  | 8.315195 | -7.61495 | 4.18E-13 | 2.68E-11 | 19.27741 |
| SCN2B | -0.98062 |  | 7.761328 | -7.61476 | 4.18E-13 | 2.68E-11 | 19.27627 |
| ATP6V1B2 | -0.8317 |  | 10.54061 | -7.61355 | 4.21E-13 | 2.69E-11 | 19.26876 |
| PCYOX1L | -0.74334 |  | 8.013302 | -7.61012 | 4.31E-13 | 2.74E-11 | 19.24742 |
| TP53INP1 | 0.720532 |  | 7.12176 | 7.586203 | 5.02E-13 | 3.15E-11 | 19.09885 |
| ZC3H7B | 0.669607 |  | 7.941652 | 7.57986 | 5.23E-13 | 3.25E-11 | 19.05951 |
| ZNF770 | 0.624289 |  | 8.831279 | 7.544409 | 6.55E-13 | 4.05E-11 | 18.83999 |
| TBC1D7 | -0.76803 |  | 7.210116 | -7.53876 | 6.79E-13 | 4.18E-11 | 18.80508 |
| ATP5C1 | -0.69685 |  | 9.909918 | -7.52667 | 7.33E-13 | 4.46E-11 | 18.73041 |
| ITGA6 | 0.731742 |  | 7.449246 | 7.526378 | 7.35E-13 | 4.46E-11 | 18.7286 |
| LRRFIP1 | 0.594889 |  | 10.26875 | 7.511082 | 8.10E-13 | 4.86E-11 | 18.63424 |
| RAB13 | 0.852811 |  | 9.334628 | 7.509717 | 8.17E-13 | 4.88E-11 | 18.62583 |
| ATP11C | 0.688246 |  | 6.672588 | 7.496654 | 8.87E-13 | 5.24E-11 | 18.54536 |
| EEF1D | 0.614942 |  | 11.13573 | 7.495443 | 8.94E-13 | 5.24E-11 | 18.5379 |
| CEBPD | 0.73278 |  | 9.977549 | 7.493305 | 9.06E-13 | 5.29E-11 | 18.52474 |
| HES1 | 0.606633 |  | 6.997821 | 7.488704 | 9.33E-13 | 5.43E-11 | 18.49643 |
| FIBP | -0.71604 |  | 8.752046 | -7.48147 | 9.77E-13 | 5.66E-11 | 18.45195 |
| SLC5A3 | 0.734278 |  | 9.287169 | 7.476758 | 1.01E-12 | 5.81E-11 | 18.42297 |
| TNFRSF10B | 0.653716 |  | 7.577139 | 7.47213 | 1.04E-12 | 5.93E-11 | 18.39452 |
| COPS3 | -0.78274 |  | 8.832175 | -7.46208 | 1.10E-12 | 6.27E-11 | 18.33282 |
| RB1 | 0.699471 |  | 8.449254 | 7.452421 | 1.17E-12 | 6.62E-11 | 18.27355 |
| C12orf10 | -0.69048 |  | 8.952177 | -7.4411 | 1.26E-12 | 7.08E-11 | 18.20413 |
| AHCYL1 | 0.600482 |  | 11.16785 | 7.434921 | 1.31E-12 | 7.28E-11 | 18.16631 |
| HIF3A | 0.665497 |  | 6.808247 | 7.433485 | 1.32E-12 | 7.32E-11 | 18.15751 |
| GRIN1 | -0.74916 |  | 7.519136 | -7.43239 | 1.33E-12 | 7.33E-11 | 18.15083 |
| TUBB3 | -0.77801 |  | 9.775171 | -7.43217 | 1.33E-12 | 7.33E-11 | 18.14946 |
| TM7SF2 | -0.91572 |  | 7.175177 | -7.41278 | 1.51E-12 | 8.15E-11 | 18.03087 |
| MYBPC1 | 0.79085 |  | 8.755681 | 7.407785 | 1.56E-12 | 8.36E-11 | 18.00034 |
| ME3 | -0.58528 |  | 7.269427 | -7.40602 | 1.57E-12 | 8.42E-11 | 17.98954 |
| GOT1 | -0.95425 |  | 9.191782 | -7.39789 | 1.66E-12 | 8.77E-11 | 17.9399 |
| MKNK2 | 0.84315 |  | 10.66267 | 7.392855 | 1.71E-12 | 9.02E-11 | 17.90921 |
| AP3M2 | -0.60984 |  | 9.277338 | -7.38678 | 1.77E-12 | 9.33E-11 | 17.87213 |
| JUND | 0.643463 |  | 11.18014 | 7.382749 | 1.82E-12 | 9.51E-11 | 17.84758 |
| HSP90B1 | 0.804647 |  | 10.33238 | 7.36794 | 2.00E-12 | 1.04E-10 | 17.75738 |
| YES1 | 0.642998 |  | 7.956164 | 7.35411 | 2.18E-12 | 1.12E-10 | 17.67326 |
| PRSS3 | -0.59792 |  | 7.848002 | -7.33984 | 2.38E-12 | 1.22E-10 | 17.5866 |
| OSBPL11 | 0.601059 |  | 8.171615 | 7.316576 | 2.75E-12 | 1.39E-10 | 17.44548 |
| C9orf64 | 0.722525 |  | 9.52032 | 7.311348 | 2.85E-12 | 1.43E-10 | 17.41381 |
| PPIH | -0.78296 |  | 7.68616 | -7.27147 | 3.65E-12 | 1.79E-10 | 17.17277 |
| SST | -1.42018 |  | 7.352859 | -7.26387 | 3.82E-12 | 1.87E-10 | 17.12698 |
| NEK7 | 0.722807 |  | 8.242596 | 7.259458 | 3.93E-12 | 1.91E-10 | 17.10035 |
| DBT | 0.635886 |  | 9.383428 | 7.25636 | 4.01E-12 | 1.94E-10 | 17.08168 |
| PRR11 | 0.767366 |  | 10.00831 | 7.252279 | 4.11E-12 | 1.98E-10 | 17.0571 |
| SF3B5 | -0.60093 |  | 9.008474 | -7.2495 | 4.18E-12 | 2.00E-10 | 17.04036 |
| SLC35B1 | -0.71426 |  | 8.26209 | -7.24059 | 4.42E-12 | 2.10E-10 | 16.98674 |
| PAK1 | -0.68665 |  | 9.637156 | -7.22307 | 4.93E-12 | 2.31E-10 | 16.88145 |
| KDELC2 | 0.880699 |  | 6.907342 | 7.221799 | 4.96E-12 | 2.32E-10 | 16.8738 |
| BAZ1A | 0.638611 |  | 6.939145 | 7.171814 | 6.76E-12 | 3.07E-10 | 16.57434 |
| KCNE4 | 0.793271 |  | 6.085269 | 7.171421 | 6.78E-12 | 3.07E-10 | 16.57199 |
| SLC1A3 | 0.684355 |  | 11.53887 | 7.161603 | 7.20E-12 | 3.25E-10 | 16.51334 |
| ZNF721 | 0.678109 |  | 10.87332 | 7.160173 | 7.26E-12 | 3.27E-10 | 16.5048 |
| TJP2 | 0.63942 |  | 7.776012 | 7.155759 | 7.46E-12 | 3.34E-10 | 16.47845 |
| GABRG2 | -0.88193 |  | 9.672083 | -7.14662 | 7.89E-12 | 3.50E-10 | 16.42393 |
| ID3 | 1.253732 |  | 7.968908 | 7.136623 | 8.39E-12 | 3.66E-10 | 16.36436 |
| UCHL1 | -0.86488 |  | 10.09115 | -7.13284 | 8.59E-12 | 3.74E-10 | 16.34181 |
| SYN2 | -0.76716 |  | 9.282273 | -7.11113 | 9.81E-12 | 4.17E-10 | 16.21268 |
| PHYHD1 | 0.911471 |  | 6.445833 | 7.107216 | 1.01E-11 | 4.26E-10 | 16.18944 |
| LOC202181 | 1.050237 |  | 8.102222 | 7.10373 | 1.03E-11 | 4.33E-10 | 16.16873 |
| CALM1 | -0.954 |  | 9.71358 | -7.10304 | 1.03E-11 | 4.34E-10 | 16.16463 |
| C21orf91 | 0.70047 |  | 8.106517 | 7.1 | 1.05E-11 | 4.41E-10 | 16.14659 |
| DHPS | -0.62516 |  | 8.072494 | -7.09386 | 1.09E-11 | 4.56E-10 | 16.11016 |
| PRELP | 0.635773 |  | 7.024953 | 7.087362 | 1.14E-11 | 4.71E-10 | 16.07162 |
| ITGAV | 0.715977 |  | 9.946483 | 7.08401 | 1.16E-11 | 4.79E-10 | 16.05175 |
| CRY1 | 0.709204 |  | 8.454042 | 7.07218 | 1.25E-11 | 5.12E-10 | 15.98168 |
| IL17RB | 0.711347 |  | 8.036969 | 7.067756 | 1.28E-11 | 5.25E-10 | 15.9555 |
| CARTPT | -0.83811 |  | 7.61914 | -7.06052 | 1.34E-11 | 5.47E-10 | 15.91272 |
| NDUFA7 | -0.81686 |  | 7.852966 | -7.05356 | 1.40E-11 | 5.69E-10 | 15.87157 |
| ADCYAP1 | -0.98315 |  | 6.072451 | -7.04476 | 1.47E-11 | 5.99E-10 | 15.81956 |
| STOM | 0.659455 |  | 10.02043 | 7.043821 | 1.48E-11 | 6.00E-10 | 15.81403 |
| ATP6V1H | -0.64945 |  | 9.007285 | -7.04234 | 1.49E-11 | 6.04E-10 | 15.80531 |
| ENO2 | -0.62256 |  | 11.0515 | -7.03113 | 1.60E-11 | 6.40E-10 | 15.73913 |
| SAMD4A | 0.626265 |  | 7.654237 | 7.021462 | 1.70E-11 | 6.75E-10 | 15.68218 |
| RWDD2B | -0.76714 |  | 7.421121 | -7.01955 | 1.72E-11 | 6.81E-10 | 15.6709 |
| FYCO1 | 0.633984 |  | 7.585254 | 7.014491 | 1.77E-11 | 6.94E-10 | 15.64113 |
| HSPA2 | 0.776341 |  | 9.684571 | 7.005312 | 1.87E-11 | 7.29E-10 | 15.58713 |
| ELF1 | 0.732973 |  | 7.189416 | 6.997156 | 1.97E-11 | 7.62E-10 | 15.53918 |
| NXN | 0.659911 |  | 8.11669 | 6.993288 | 2.01E-11 | 7.71E-10 | 15.51645 |
| TMEM35 | -0.66864 |  | 7.635432 | -6.98317 | 2.14E-11 | 8.17E-10 | 15.45705 |
| SLC1A6 | -0.8164 |  | 6.235737 | -6.98134 | 2.16E-11 | 8.24E-10 | 15.44634 |
| PSD2 | 0.650868 |  | 9.798048 | 6.964068 | 2.40E-11 | 9.03E-10 | 15.34506 |
| SURF2 | -0.85645 |  | 6.351404 | -6.96161 | 2.44E-11 | 9.09E-10 | 15.33066 |
| FOXD1 | 1.126 |  | 5.920588 | 6.953451 | 2.56E-11 | 9.51E-10 | 15.28292 |
| ITGB5 | 0.710975 |  | 8.484325 | 6.9465 | 2.67E-11 | 9.89E-10 | 15.24226 |
| ZNF217 | 0.835732 |  | 6.660918 | 6.94038 | 2.77E-11 | 1.02E-09 | 15.20649 |
| RPH3A | -0.80219 |  | 8.531333 | -6.93989 | 2.78E-11 | 1.02E-09 | 15.20366 |
| SLC39A12 | 0.965916 |  | 8.290933 | 6.935027 | 2.86E-11 | 1.04E-09 | 15.17522 |
| MAP4K4 | 0.598357 |  | 9.51881 | 6.931429 | 2.92E-11 | 1.06E-09 | 15.15422 |
| SDHB | -0.68957 |  | 7.885093 | -6.92496 | 3.04E-11 | 1.10E-09 | 15.11649 |
| DHRS7B | -1.13752 |  | 6.586617 | -6.91776 | 3.18E-11 | 1.15E-09 | 15.07446 |
| MAML2 | 0.64066 |  | 7.342559 | 6.916035 | 3.21E-11 | 1.16E-09 | 15.06442 |
| ZDHHC23 | -0.67438 |  | 8.059126 | -6.91275 | 3.27E-11 | 1.18E-09 | 15.04529 |
| PRMT7 | -1.03719 |  | 6.573616 | -6.89562 | 3.63E-11 | 1.29E-09 | 14.94553 |
| ZNF566 | 0.860629 |  | 6.531333 | 6.891839 | 3.71E-11 | 1.31E-09 | 14.92356 |
| EFHD1 | 0.718514 |  | 10.34393 | 6.885652 | 3.85E-11 | 1.35E-09 | 14.8876 |
| MDH1 | -0.85875 |  | 11.97532 | -6.87606 | 4.08E-11 | 1.42E-09 | 14.83192 |
| NUDT18 | -0.88673 |  | 6.75149 | -6.87282 | 4.16E-11 | 1.44E-09 | 14.81311 |
| SKI | 0.611632 |  | 8.44868 | 6.869706 | 4.24E-11 | 1.46E-09 | 14.79502 |
| ANXA6 | -0.66024 |  | 7.682703 | -6.85695 | 4.57E-11 | 1.57E-09 | 14.72109 |
| AMPH | -0.9087 |  | 9.173397 | -6.85533 | 4.62E-11 | 1.58E-09 | 14.7117 |
| WDR70 | -0.75963 |  | 7.335866 | -6.83621 | 5.18E-11 | 1.75E-09 | 14.60102 |
| PSMA5 | -0.63343 |  | 9.636798 | -6.8295 | 5.39E-11 | 1.81E-09 | 14.56228 |
| SERPINE2 | 0.606772 |  | 10.63953 | 6.828503 | 5.42E-11 | 1.81E-09 | 14.5565 |
| PBXIP1 | 0.693415 |  | 8.525372 | 6.825968 | 5.50E-11 | 1.84E-09 | 14.54186 |
| EEF1A2 | -0.85884 |  | 9.518127 | -6.82034 | 5.69E-11 | 1.89E-09 | 14.50936 |
| RASEF | 0.820608 |  | 9.266199 | 6.799493 | 6.44E-11 | 2.12E-09 | 14.38918 |
| SLC7A11 | 0.668087 |  | 7.878259 | 6.796478 | 6.56E-11 | 2.15E-09 | 14.37182 |
| MSX1 | 0.709428 |  | 7.852185 | 6.788363 | 6.88E-11 | 2.25E-09 | 14.32512 |
| FAM107A | 0.767378 |  | 11.69629 | 6.785848 | 6.99E-11 | 2.28E-09 | 14.31065 |
| XRCC2 | 0.688906 |  | 8.745567 | 6.775832 | 7.41E-11 | 2.38E-09 | 14.25308 |
| FAM63A | 0.796225 |  | 9.50045 | 6.772837 | 7.55E-11 | 2.41E-09 | 14.23587 |
| LEF1 | 0.647386 |  | 7.188581 | 6.772564 | 7.56E-11 | 2.41E-09 | 14.23431 |
| ZFP36 | 0.835168 |  | 8.794784 | 6.764562 | 7.93E-11 | 2.52E-09 | 14.18837 |
| TNS1 | 0.764618 |  | 8.679895 | 6.75327 | 8.48E-11 | 2.67E-09 | 14.12361 |
| GNG3 | -0.76703 |  | 9.488366 | -6.73938 | 9.20E-11 | 2.88E-09 | 14.04407 |
| HAGH | -0.59546 |  | 9.860969 | -6.72514 | 1.00E-10 | 3.10E-09 | 13.96265 |
| AEBP1 | 0.921925 |  | 8.302087 | 6.717104 | 1.05E-10 | 3.25E-09 | 13.91673 |
| NVL | -1.05071 |  | 6.410156 | -6.71379 | 1.07E-10 | 3.29E-09 | 13.89782 |
| CD200 | -0.76695 |  | 8.131918 | -6.70253 | 1.14E-10 | 3.49E-09 | 13.83361 |
| MXI1 | 0.623354 |  | 10.10843 | 6.699609 | 1.16E-10 | 3.54E-09 | 13.81694 |
| ZNF423 | 0.722198 |  | 8.120862 | 6.687859 | 1.25E-10 | 3.77E-09 | 13.75002 |
| SLAIN1 | 0.648025 |  | 9.486976 | 6.685414 | 1.26E-10 | 3.82E-09 | 13.73611 |
| LARGE | -0.65371 |  | 8.254861 | -6.67675 | 1.33E-10 | 4.01E-09 | 13.68684 |
| PTCHD1 | 0.644623 |  | 7.859699 | 6.670714 | 1.38E-10 | 4.13E-09 | 13.65253 |
| MYO10 | 0.683614 |  | 8.645224 | 6.667247 | 1.41E-10 | 4.21E-09 | 13.63284 |
| HMGCLL1 | -0.59332 |  | 6.53255 | -6.65351 | 1.53E-10 | 4.52E-09 | 13.55491 |
| CRMP1 | -0.62729 |  | 9.298463 | -6.65254 | 1.53E-10 | 4.54E-09 | 13.54938 |
| TNS3 | 0.813661 |  | 10.08786 | 6.637677 | 1.67E-10 | 4.91E-09 | 13.46518 |
| CASP7 | 0.758981 |  | 7.314205 | 6.635022 | 1.70E-10 | 4.98E-09 | 13.45015 |
| PTPN3 | -0.71423 |  | 6.364162 | -6.63089 | 1.74E-10 | 5.07E-09 | 13.42678 |
| ZBTB20 | 0.707148 |  | 9.895881 | 6.620464 | 1.85E-10 | 5.36E-09 | 13.36784 |
| BEX5 | -0.71693 |  | 10.7846 | -6.6181 | 1.88E-10 | 5.42E-09 | 13.3545 |
| ZBTB41 | 0.622578 |  | 9.15469 | 6.605775 | 2.02E-10 | 5.79E-09 | 13.28491 |
| KCNJ10 | 0.725781 |  | 8.316526 | 6.596269 | 2.13E-10 | 6.10E-09 | 13.23131 |
| ANGPT1 | 0.6077 |  | 6.96744 | 6.591125 | 2.20E-10 | 6.26E-09 | 13.20233 |
| HINT3 | 0.596957 |  | 7.887439 | 6.5832 | 2.30E-10 | 6.51E-09 | 13.15772 |
| DIRAS1 | -0.6191 |  | 7.075197 | -6.58319 | 2.30E-10 | 6.51E-09 | 13.15766 |
| CDC42EP4 | 0.58508 |  | 8.783731 | 6.576429 | 2.39E-10 | 6.73E-09 | 13.11963 |
| SNCA | -0.59896 |  | 10.45902 | -6.56042 | 2.63E-10 | 7.33E-09 | 13.02971 |
| SUSD4 | -0.63503 |  | 7.853207 | -6.54862 | 2.81E-10 | 7.81E-09 | 12.96349 |
| ADAM33 | 0.763275 |  | 7.933522 | 6.547797 | 2.82E-10 | 7.83E-09 | 12.95889 |
| GBP1 | 0.666159 |  | 6.814551 | 6.54578 | 2.86E-10 | 7.91E-09 | 12.94759 |
| GLS2 | -0.65644 |  | 6.77179 | -6.53089 | 3.12E-10 | 8.52E-09 | 12.8642 |
| SGIP1 | -0.86064 |  | 8.464128 | -6.52777 | 3.17E-10 | 8.65E-09 | 12.84675 |
| GABRA1 | -0.84754 |  | 8.677107 | -6.5261 | 3.20E-10 | 8.72E-09 | 12.83742 |
| CNTNAP5 | -0.63971 |  | 6.643946 | -6.52079 | 3.30E-10 | 8.97E-09 | 12.80772 |
| SULT4A1 | -0.73984 |  | 9.148189 | -6.51871 | 3.34E-10 | 9.06E-09 | 12.79614 |
| HEY2 | 0.653127 |  | 7.156817 | 6.500695 | 3.71E-10 | 9.89E-09 | 12.69557 |
| GLUL | 0.608585 |  | 11.03342 | 6.485495 | 4.05E-10 | 1.07E-08 | 12.61089 |
| GABRG1 | 0.696482 |  | 7.856607 | 6.48488 | 4.06E-10 | 1.08E-08 | 12.60747 |
| UBL7 | -0.8998 |  | 7.356692 | -6.4844 | 4.07E-10 | 1.08E-08 | 12.60482 |
| NUDT2 | -0.91496 |  | 6.995496 | -6.47054 | 4.41E-10 | 1.16E-08 | 12.5277 |
| ATP1A1 | -0.64222 |  | 10.97024 | -6.46975 | 4.43E-10 | 1.16E-08 | 12.52332 |
| PDK4 | 0.74558 |  | 8.688474 | 6.454952 | 4.83E-10 | 1.24E-08 | 12.44118 |
| CHCHD6 | -0.75055 |  | 7.985894 | -6.45419 | 4.85E-10 | 1.25E-08 | 12.43698 |
| MECR | -0.8398 |  | 6.576607 | -6.43194 | 5.51E-10 | 1.39E-08 | 12.31369 |
| MT1X | 0.744627 |  | 10.97939 | 6.423291 | 5.78E-10 | 1.45E-08 | 12.26588 |
| ZNRF3 | 0.699528 |  | 8.520367 | 6.410418 | 6.23E-10 | 1.56E-08 | 12.19478 |
| RNF181 | -0.73112 |  | 8.116357 | -6.40988 | 6.25E-10 | 1.56E-08 | 12.19181 |
| HSPB3 | -0.98387 |  | 6.275791 | -6.39651 | 6.74E-10 | 1.67E-08 | 12.11811 |
| SPP1 | 0.726918 |  | 9.957081 | 6.394593 | 6.82E-10 | 1.68E-08 | 12.10753 |
| TNFRSF1A | 0.760626 |  | 8.481023 | 6.390812 | 6.96E-10 | 1.71E-08 | 12.0867 |
| NECAP1 | -0.7762 |  | 9.250937 | -6.38947 | 7.02E-10 | 1.72E-08 | 12.07929 |
| SCRIB | 0.646882 |  | 9.060527 | 6.375791 | 7.59E-10 | 1.85E-08 | 12.00407 |
| NIT2 | -0.71071 |  | 7.774071 | -6.35905 | 8.34E-10 | 2.00E-08 | 11.91213 |
| CSRP1 | 0.599042 |  | 11.27385 | 6.355677 | 8.50E-10 | 2.03E-08 | 11.89364 |
| GABRA4 | -0.65686 |  | 7.358998 | -6.35015 | 8.78E-10 | 2.09E-08 | 11.86332 |
| GAD2 | -0.62163 |  | 7.145216 | -6.34683 | 8.94E-10 | 2.12E-08 | 11.84515 |
| CAPNS1 | -0.7296 |  | 9.305984 | -6.34625 | 8.97E-10 | 2.12E-08 | 11.84197 |
| GPAM | 0.673181 |  | 8.54522 | 6.346241 | 8.97E-10 | 2.12E-08 | 11.84193 |
| PGF | 0.77027 |  | 9.303717 | 6.33533 | 9.54E-10 | 2.24E-08 | 11.7822 |
| DTX3L | 0.670201 |  | 7.267777 | 6.320536 | 1.04E-09 | 2.43E-08 | 11.70133 |
| ARPC1A | -1.08607 |  | 8.440797 | -6.31471 | 1.07E-09 | 2.50E-08 | 11.66951 |
| TRUB2 | -0.65948 |  | 8.024393 | -6.31349 | 1.08E-09 | 2.50E-08 | 11.66285 |
| CMTM1 | -0.63376 |  | 6.094187 | -6.30271 | 1.15E-09 | 2.63E-08 | 11.60408 |
| RNF175 | -0.64743 |  | 8.817445 | -6.29897 | 1.17E-09 | 2.68E-08 | 11.5837 |
| SV2B | -0.72149 |  | 9.947098 | -6.29848 | 1.18E-09 | 2.68E-08 | 11.58105 |
| MT1F | 0.723337 |  | 10.74463 | 6.297754 | 1.18E-09 | 2.69E-08 | 11.57708 |
| CLEC2L | -0.78857 |  | 7.111334 | -6.29076 | 1.23E-09 | 2.77E-08 | 11.53901 |
| MAEL | -0.83006 |  | 5.662931 | -6.27269 | 1.36E-09 | 3.05E-08 | 11.44076 |
| PPM1E | -0.8623 |  | 9.068824 | -6.26803 | 1.40E-09 | 3.12E-08 | 11.41549 |
| ANKRD13D | 0.586666 |  | 8.18183 | 6.264863 | 1.42E-09 | 3.17E-08 | 11.39829 |
| TTLL1 | -0.73832 |  | 7.136585 | -6.26206 | 1.44E-09 | 3.22E-08 | 11.38307 |
| KCNJ16 | 0.732724 |  | 7.52715 | 6.260773 | 1.45E-09 | 3.24E-08 | 11.3761 |
| RAD51C | -0.62127 |  | 7.431922 | -6.26017 | 1.46E-09 | 3.24E-08 | 11.37284 |
| FNBP1L | 0.621831 |  | 8.335146 | 6.256937 | 1.49E-09 | 3.30E-08 | 11.35531 |
| SPHKAP | -1.15992 |  | 8.871482 | -6.24896 | 1.55E-09 | 3.44E-08 | 11.3121 |
| TMEM151A | -0.95118 |  | 6.75955 | -6.24841 | 1.56E-09 | 3.44E-08 | 11.3091 |
| RAB3C | -0.75118 |  | 7.159038 | -6.24536 | 1.58E-09 | 3.49E-08 | 11.29259 |
| CCK | -0.9741 |  | 9.502231 | -6.2448 | 1.59E-09 | 3.50E-08 | 11.28959 |
| COL5A3 | 0.624681 |  | 7.631575 | 6.232324 | 1.70E-09 | 3.72E-08 | 11.2221 |
| MPV17 | -0.82707 |  | 7.774675 | -6.22909 | 1.74E-09 | 3.78E-08 | 11.20462 |
| RASL12 | 0.938133 |  | 7.196898 | 6.225156 | 1.77E-09 | 3.86E-08 | 11.18338 |
| SERPINA3 | 1.210227 |  | 9.05378 | 6.22509 | 1.78E-09 | 3.86E-08 | 11.18302 |
| SLCO4A1 | 0.81818 |  | 7.326186 | 6.222546 | 1.80E-09 | 3.89E-08 | 11.16929 |
| PRKX | 0.796064 |  | 6.872379 | 6.214599 | 1.88E-09 | 4.06E-08 | 11.12641 |
| CACNG3 | -0.7224 |  | 8.60644 | -6.20788 | 1.95E-09 | 4.20E-08 | 11.09021 |
| TUBA4A | -0.63678 |  | 11.23582 | -6.20431 | 1.99E-09 | 4.26E-08 | 11.07094 |
| ANKRD39 | -0.76176 |  | 7.081605 | -6.20328 | 2.01E-09 | 4.27E-08 | 11.06539 |
| IFI16 | 0.590271 |  | 8.170803 | 6.195945 | 2.09E-09 | 4.42E-08 | 11.02593 |
| FIG4 | -0.62159 |  | 9.253588 | -6.19191 | 2.14E-09 | 4.51E-08 | 11.00422 |
| STAT4 | -0.85053 |  | 7.968855 | -6.18961 | 2.16E-09 | 4.55E-08 | 10.99187 |
| CLDN15 | 0.593509 |  | 6.575414 | 6.185804 | 2.21E-09 | 4.64E-08 | 10.9714 |
| RAB3B | -0.58568 |  | 6.111062 | -6.17721 | 2.32E-09 | 4.83E-08 | 10.92525 |
| STMN2 | -0.78204 |  | 10.92846 | -6.17382 | 2.36E-09 | 4.92E-08 | 10.90703 |
| SMYD3 | -0.69945 |  | 8.032894 | -6.1705 | 2.41E-09 | 4.98E-08 | 10.88926 |
| C1QTNF4 | -1.17461 |  | 6.352483 | -6.16165 | 2.53E-09 | 5.21E-08 | 10.8418 |
| PPAP2B | 0.585865 |  | 9.326674 | 6.159989 | 2.55E-09 | 5.23E-08 | 10.8329 |
| COLEC12 | 0.766165 |  | 7.447208 | 6.155032 | 2.62E-09 | 5.36E-08 | 10.80635 |
| TMEM141 | -0.68608 |  | 7.756026 | -6.15116 | 2.68E-09 | 5.47E-08 | 10.78561 |
| MAP2K4 | -0.59036 |  | 9.452679 | -6.14461 | 2.78E-09 | 5.66E-08 | 10.75061 |
| CNOT10 | -0.79104 |  | 6.550963 | -6.1428 | 2.81E-09 | 5.71E-08 | 10.7409 |
| BEX4 | -0.62122 |  | 9.981387 | -6.13807 | 2.88E-09 | 5.84E-08 | 10.71565 |
| CD163 | 0.799596 |  | 6.783309 | 6.114146 | 3.29E-09 | 6.57E-08 | 10.58801 |
| MAST3 | -0.67012 |  | 10.36881 | -6.11191 | 3.33E-09 | 6.64E-08 | 10.57612 |
| CHST6 | 1.099349 |  | 6.326281 | 6.108935 | 3.39E-09 | 6.70E-08 | 10.56027 |
| MRPL15 | -0.63257 |  | 8.475129 | -6.10869 | 3.39E-09 | 6.70E-08 | 10.55898 |
| FKBP1B | -0.59194 |  | 9.908083 | -6.10282 | 3.50E-09 | 6.90E-08 | 10.52771 |
| GPRC5B | 0.592791 |  | 10.05865 | 6.094079 | 3.67E-09 | 7.20E-08 | 10.48126 |
| CDC37 | -0.89536 |  | 7.860623 | -6.08813 | 3.80E-09 | 7.42E-08 | 10.44964 |
| EXOSC5 | -0.83268 |  | 6.60685 | -6.07823 | 4.01E-09 | 7.79E-08 | 10.39714 |
| CALB1 | -0.69354 |  | 7.552508 | -6.067 | 4.26E-09 | 8.25E-08 | 10.33763 |
| PCP4 | -0.79969 |  | 9.600153 | -6.04767 | 4.74E-09 | 9.06E-08 | 10.2354 |
| FBXW12 | 0.621603 |  | 9.355684 | 6.030992 | 5.20E-09 | 9.83E-08 | 10.14737 |
| CAMK1G | -0.7693 |  | 6.898179 | -6.02064 | 5.50E-09 | 1.03E-07 | 10.09285 |
| PCSK1 | -0.97043 |  | 7.191968 | -6.0158 | 5.65E-09 | 1.05E-07 | 10.06736 |
| SDC4 | 0.726114 |  | 9.531655 | 6.013573 | 5.71E-09 | 1.06E-07 | 10.05565 |
| ALAS1 | -0.65425 |  | 7.81488 | -6.00267 | 6.06E-09 | 1.12E-07 | 9.998342 |
| EPS8 | 0.662385 |  | 7.837361 | 5.976038 | 7.01E-09 | 1.26E-07 | 9.8587 |
| PSMD8 | -0.97516 |  | 8.457591 | -5.9748 | 7.06E-09 | 1.27E-07 | 9.852232 |
| BCAR3 | 0.638416 |  | 7.205368 | 5.967591 | 7.34E-09 | 1.31E-07 | 9.81451 |
| GPR22 | -0.67451 |  | 7.026493 | -5.96199 | 7.57E-09 | 1.35E-07 | 9.785234 |
| GBP2 | 0.650471 |  | 7.888011 | 5.961442 | 7.59E-09 | 1.35E-07 | 9.782369 |
| RAB31 | 0.612316 |  | 10.80467 | 5.955232 | 7.85E-09 | 1.39E-07 | 9.749935 |
| ID2 | 0.59072 |  | 9.953082 | 5.946667 | 8.22E-09 | 1.46E-07 | 9.705249 |
| KLF4 | 0.795364 |  | 7.059626 | 5.943004 | 8.39E-09 | 1.48E-07 | 9.686151 |
| LAMB1 | -0.68201 |  | 7.29344 | -5.93158 | 8.92E-09 | 1.56E-07 | 9.626662 |
| ATOH7 | -0.66261 |  | 6.008908 | -5.92973 | 9.01E-09 | 1.57E-07 | 9.617011 |
| EBF1 | 0.651832 |  | 6.560105 | 5.927023 | 9.14E-09 | 1.59E-07 | 9.602941 |
| SLC25A18 | 0.771361 |  | 10.05301 | 5.921702 | 9.41E-09 | 1.62E-07 | 9.57527 |
| DDIT4L | 0.804529 |  | 7.852735 | 5.917287 | 9.64E-09 | 1.66E-07 | 9.55233 |
| SEMA3F | 0.716847 |  | 7.599265 | 5.916785 | 9.66E-09 | 1.66E-07 | 9.549721 |
| TRAPPC1 | -0.85946 |  | 7.198795 | -5.90825 | 1.01E-08 | 1.74E-07 | 9.505435 |
| PSMC4 | -0.71256 |  | 7.697162 | -5.90543 | 1.03E-08 | 1.76E-07 | 9.490783 |
| MAL2 | -0.85008 |  | 8.275799 | -5.9035 | 1.04E-08 | 1.77E-07 | 9.480799 |
| NECAB1 | -0.80194 |  | 7.102633 | -5.895 | 1.09E-08 | 1.84E-07 | 9.436735 |
| RAB11FIP3 | 0.644757 |  | 10.03393 | 5.883383 | 1.16E-08 | 1.95E-07 | 9.376594 |
| SSSCA1 | -0.89722 |  | 5.906971 | -5.88199 | 1.17E-08 | 1.96E-07 | 9.369398 |
| PMAIP1 | 0.718733 |  | 5.678267 | 5.877089 | 1.20E-08 | 2.00E-07 | 9.344056 |
| RHPN2 | 0.695054 |  | 8.077601 | 5.874922 | 1.21E-08 | 2.02E-07 | 9.332862 |
| RAB20 | 0.608984 |  | 6.270247 | 5.872148 | 1.23E-08 | 2.05E-07 | 9.318533 |
| FOXC1 | 0.896392 |  | 8.59237 | 5.869008 | 1.25E-08 | 2.08E-07 | 9.30232 |
| SNX31 | 1.072642 |  | 5.879478 | 5.867413 | 1.26E-08 | 2.09E-07 | 9.29409 |
| NAPA | -0.61163 |  | 7.394537 | -5.85685 | 1.33E-08 | 2.20E-07 | 9.239606 |
| UQCRC1 | -0.8276 |  | 9.384899 | -5.84574 | 1.42E-08 | 2.32E-07 | 9.182419 |
| DMAP1 | -0.86001 |  | 7.359779 | -5.84148 | 1.45E-08 | 2.37E-07 | 9.160456 |
| CECR6 | -0.7538 |  | 7.392163 | -5.83128 | 1.53E-08 | 2.48E-07 | 9.108061 |
| ELMO1 | -0.6411 |  | 7.958663 | -5.82466 | 1.58E-08 | 2.55E-07 | 9.074056 |
| SYT13 | -0.65751 |  | 9.737876 | -5.81941 | 1.63E-08 | 2.62E-07 | 9.047116 |
| ATP6AP1 | -0.69134 |  | 10.05509 | -5.81723 | 1.65E-08 | 2.64E-07 | 9.035962 |
| HBQ1 | -0.81959 |  | 5.636543 | -5.81263 | 1.69E-08 | 2.70E-07 | 9.012395 |
| NELL1 | -0.80422 |  | 7.487384 | -5.80724 | 1.74E-08 | 2.78E-07 | 8.984755 |
| KIFAP3 | -0.78524 |  | 9.137312 | -5.80702 | 1.74E-08 | 2.78E-07 | 8.983664 |
| GAD1 | -0.71439 |  | 8.81761 | -5.79265 | 1.88E-08 | 2.96E-07 | 8.910129 |
| IGFBP7 | 0.641705 |  | 10.34586 | 5.789686 | 1.91E-08 | 3.00E-07 | 8.89501 |
| PSMC3 | -0.75431 |  | 8.001315 | -5.782 | 1.99E-08 | 3.11E-07 | 8.855776 |
| RIT2 | -0.7346 |  | 7.713947 | -5.78194 | 1.99E-08 | 3.11E-07 | 8.855447 |
| SLC27A4 | -0.73475 |  | 6.680479 | -5.78154 | 1.99E-08 | 3.11E-07 | 8.853414 |
| ANKRD36B | 0.774133 |  | 10.21255 | 5.768956 | 2.13E-08 | 3.30E-07 | 8.789277 |
| TFPT | -0.66274 |  | 8.035925 | -5.75471 | 2.30E-08 | 3.53E-07 | 8.716805 |
| RARRES2 | 0.862561 |  | 7.162036 | 5.712113 | 2.87E-08 | 4.34E-07 | 8.500876 |
| CGREF1 | -0.79737 |  | 6.421898 | -5.71102 | 2.89E-08 | 4.36E-07 | 8.495374 |
| ACTL6B | -0.84798 |  | 7.420681 | -5.70921 | 2.92E-08 | 4.39E-07 | 8.486217 |
| SLC14A1 | 0.793865 |  | 8.364411 | 5.690675 | 3.22E-08 | 4.77E-07 | 8.39269 |
| ORAI3 | 0.590366 |  | 7.307493 | 5.688574 | 3.25E-08 | 4.81E-07 | 8.382105 |
| TMEM158 | -0.82288 |  | 8.727809 | -5.68723 | 3.28E-08 | 4.84E-07 | 8.375359 |
| DDX25 | -0.60241 |  | 7.798456 | -5.67782 | 3.44E-08 | 5.04E-07 | 8.327981 |
| KLHDC8A | -0.61795 |  | 7.219652 | -5.67624 | 3.47E-08 | 5.08E-07 | 8.320034 |
| VASP | 0.687746 |  | 6.964116 | 5.658006 | 3.82E-08 | 5.50E-07 | 8.228442 |
| TRIM17 | -0.73087 |  | 5.516096 | -5.64638 | 4.05E-08 | 5.80E-07 | 8.17016 |
| KCTD4 | -0.65538 |  | 6.995573 | -5.64344 | 4.12E-08 | 5.88E-07 | 8.155433 |
| TMOD1 | -0.61812 |  | 8.62508 | -5.64028 | 4.19E-08 | 5.94E-07 | 8.13965 |
| F3 | 0.716767 |  | 8.789581 | 5.636002 | 4.28E-08 | 6.05E-07 | 8.118236 |
| GABRA5 | -0.59937 |  | 6.667284 | -5.63055 | 4.40E-08 | 6.21E-07 | 8.090967 |
| MXRA8 | 0.613919 |  | 6.728315 | 5.626547 | 4.50E-08 | 6.31E-07 | 8.070985 |
| GPR4 | 0.603732 |  | 6.362475 | 5.624041 | 4.55E-08 | 6.37E-07 | 8.058474 |
| NRIP3 | -0.65008 |  | 10.32015 | -5.62153 | 4.61E-08 | 6.43E-07 | 8.045944 |
| OLFM3 | -0.67468 |  | 7.592001 | -5.61223 | 4.84E-08 | 6.71E-07 | 7.999568 |
| RGS7 | -0.62325 |  | 8.986441 | -5.61137 | 4.86E-08 | 6.72E-07 | 7.995253 |
| PLXNB1 | 0.679527 |  | 6.731051 | 5.610118 | 4.90E-08 | 6.76E-07 | 7.989036 |
| ITGA10 | 0.625634 |  | 7.124325 | 5.59496 | 5.30E-08 | 7.26E-07 | 7.913589 |
| KLF2 | 0.595283 |  | 8.83224 | 5.591285 | 5.40E-08 | 7.38E-07 | 7.895322 |
| MAP7D2 | -0.74033 |  | 9.655115 | -5.58746 | 5.51E-08 | 7.52E-07 | 7.8763 |
| GRM1 | -0.61481 |  | 7.229172 | -5.5803 | 5.71E-08 | 7.77E-07 | 7.840757 |
| SMYD5 | -0.72271 |  | 6.817312 | -5.576 | 5.84E-08 | 7.92E-07 | 7.819435 |
| VPS28 | -0.65796 |  | 9.031188 | -5.56652 | 6.14E-08 | 8.27E-07 | 7.772461 |
| RBP4 | -0.76367 |  | 6.895634 | -5.55665 | 6.46E-08 | 8.66E-07 | 7.723661 |
| SERPINB6 | 0.589822 |  | 8.708156 | 5.546435 | 6.80E-08 | 9.06E-07 | 7.673162 |
| HMP19 | -0.66701 |  | 10.1002 | -5.54341 | 6.91E-08 | 9.19E-07 | 7.658209 |
| HEPH | 0.678138 |  | 8.026632 | 5.539656 | 7.05E-08 | 9.33E-07 | 7.63971 |
| MAD2L1BP | -0.5906 |  | 7.315847 | -5.53665 | 7.16E-08 | 9.46E-07 | 7.624887 |
| RTBDN | -0.75872 |  | 5.980523 | -5.53171 | 7.34E-08 | 9.68E-07 | 7.600558 |
| LEAP2 | 0.708388 |  | 6.597111 | 5.522816 | 7.68E-08 | 1.01E-06 | 7.556743 |
| MRGPRF | 0.797331 |  | 6.179137 | 5.503724 | 8.47E-08 | 1.10E-06 | 7.462925 |
| DNTTIP1 | -0.66965 |  | 7.079369 | -5.50309 | 8.50E-08 | 1.10E-06 | 7.459829 |
| CDK5 | -0.79241 |  | 8.214582 | -5.49961 | 8.65E-08 | 1.12E-06 | 7.442764 |
| MAP2K1 | -0.59823 |  | 9.943757 | -5.4959 | 8.82E-08 | 1.14E-06 | 7.424573 |
| CDK7 | -0.81786 |  | 7.706118 | -5.47029 | 1.00E-07 | 1.28E-06 | 7.299277 |
| ZNF486 | 0.636844 |  | 8.125125 | 5.466076 | 1.03E-07 | 1.30E-06 | 7.278689 |
| NFIC | 0.627416 |  | 9.330415 | 5.464058 | 1.04E-07 | 1.31E-06 | 7.26884 |
| LATS2 | 0.646862 |  | 7.568956 | 5.460206 | 1.06E-07 | 1.33E-06 | 7.250055 |
| GABRD | -0.89246 |  | 6.920286 | -5.45857 | 1.07E-07 | 1.34E-06 | 7.242061 |
| ASNS | -0.59234 |  | 9.462379 | -5.4307 | 1.23E-07 | 1.52E-06 | 7.106514 |
| RTN4IP1 | -0.77337 |  | 6.651913 | -5.4146 | 1.33E-07 | 1.64E-06 | 7.02842 |
| APOO | -0.66336 |  | 8.697591 | -5.40657 | 1.39E-07 | 1.70E-06 | 6.989567 |
| CRB2 | 0.778783 |  | 5.683641 | 5.402026 | 1.42E-07 | 1.73E-06 | 6.967581 |
| HDAC1 | 0.630568 |  | 9.078532 | 5.401049 | 1.43E-07 | 1.74E-06 | 6.962863 |
| TRIM47 | 0.710963 |  | 7.315157 | 5.400499 | 1.43E-07 | 1.74E-06 | 6.960203 |
| PLK2 | -0.77644 |  | 8.693634 | -5.39691 | 1.46E-07 | 1.77E-06 | 6.942871 |
| NAT6 | -0.75122 |  | 6.00307 | -5.39598 | 1.46E-07 | 1.77E-06 | 6.938381 |
| ZFPM2 | -0.66418 |  | 8.029788 | -5.39568 | 1.47E-07 | 1.77E-06 | 6.936905 |
| C11orf73 | -0.61128 |  | 7.634468 | -5.38825 | 1.52E-07 | 1.83E-06 | 6.901058 |
| NLRC5 | 0.71469 |  | 7.173979 | 5.387364 | 1.53E-07 | 1.84E-06 | 6.896785 |
| HAS1 | -0.7032 |  | 6.577455 | -5.38637 | 1.54E-07 | 1.85E-06 | 6.892012 |
| IFI6 | -0.65104 |  | 8.785298 | -5.38422 | 1.55E-07 | 1.87E-06 | 6.881605 |
| PPEF1 | -0.85084 |  | 5.989203 | -5.37133 | 1.66E-07 | 1.98E-06 | 6.819558 |
| GLRX | -0.6611 |  | 9.329504 | -5.36223 | 1.73E-07 | 2.07E-06 | 6.775765 |
| SOSTDC1 | -0.95919 |  | 6.435205 | -5.36139 | 1.74E-07 | 2.07E-06 | 6.771759 |
| SLITRK4 | -0.58695 |  | 8.982797 | -5.35243 | 1.82E-07 | 2.15E-06 | 6.72871 |
| BDNF | -0.65733 |  | 6.666767 | -5.35034 | 1.84E-07 | 2.17E-06 | 6.718681 |
| SYTL5 | -0.68973 |  | 6.488182 | -5.32641 | 2.08E-07 | 2.40E-06 | 6.604122 |
| ARHGDIG | -0.62939 |  | 7.266268 | -5.31993 | 2.14E-07 | 2.47E-06 | 6.573162 |
| ZIC1 | 0.780226 |  | 7.904941 | 5.31188 | 2.23E-07 | 2.56E-06 | 6.534749 |
| DUSP4 | -0.71888 |  | 6.458162 | -5.30428 | 2.32E-07 | 2.64E-06 | 6.498543 |
| NRGN | -0.65439 |  | 10.57264 | -5.30309 | 2.33E-07 | 2.65E-06 | 6.492877 |
| AAAS | -0.6215 |  | 6.200252 | -5.30176 | 2.35E-07 | 2.67E-06 | 6.486511 |
| HPRT1 | -0.6467 |  | 9.400851 | -5.30027 | 2.36E-07 | 2.68E-06 | 6.47942 |
| PODXL2 | -0.74484 |  | 7.178958 | -5.27408 | 2.69E-07 | 3.03E-06 | 6.35501 |
| GPS1 | -0.63554 |  | 7.298197 | -5.27197 | 2.72E-07 | 3.06E-06 | 6.345007 |
| WDR46 | -0.77638 |  | 6.628418 | -5.27176 | 2.72E-07 | 3.06E-06 | 6.344019 |
| BCKDK | -0.61549 |  | 7.743963 | -5.27153 | 2.73E-07 | 3.06E-06 | 6.34292 |
| APOLD1 | 0.690102 |  | 9.613772 | 5.266805 | 2.79E-07 | 3.12E-06 | 6.320537 |
| HLA-DRB4 | 0.843793 |  | 5.8109 | 5.264919 | 2.82E-07 | 3.15E-06 | 6.311608 |
| LPAR4 | 0.677796 |  | 6.333242 | 5.259386 | 2.89E-07 | 3.23E-06 | 6.285424 |
| TCF7L1 | 0.659839 |  | 6.030689 | 5.24783 | 3.06E-07 | 3.41E-06 | 6.230809 |
| FXYD7 | -0.65907 |  | 7.508007 | -5.24581 | 3.10E-07 | 3.44E-06 | 6.22129 |
| BLVRB | -0.71797 |  | 8.085907 | -5.24457 | 3.11E-07 | 3.45E-06 | 6.215437 |
| PRDM16 | 0.605591 |  | 7.190013 | 5.240068 | 3.18E-07 | 3.52E-06 | 6.194183 |
| FHOD3 | -0.64907 |  | 7.567364 | -5.23736 | 3.23E-07 | 3.56E-06 | 6.181392 |
| ANKRD34C | -0.64821 |  | 7.096675 | -5.23572 | 3.25E-07 | 3.58E-06 | 6.173672 |
| RRAGB | -0.8685 |  | 6.061356 | -5.23398 | 3.28E-07 | 3.61E-06 | 6.165466 |
| ZCCHC12 | -0.62174 |  | 7.891962 | -5.22821 | 3.38E-07 | 3.71E-06 | 6.138324 |
| SERPINF1 | -0.76606 |  | 7.612853 | -5.21759 | 3.56E-07 | 3.87E-06 | 6.088379 |
| TFAP2C | 0.610632 |  | 5.582755 | 5.208081 | 3.73E-07 | 4.03E-06 | 6.043701 |
| VSNL1 | -0.65986 |  | 11.41627 | -5.20106 | 3.86E-07 | 4.17E-06 | 6.01079 |
| DOCK3 | -0.67616 |  | 7.934692 | -5.19255 | 4.02E-07 | 4.32E-06 | 5.970915 |
| RALYL | -0.58671 |  | 9.573211 | -5.16634 | 4.57E-07 | 4.88E-06 | 5.848477 |
| UBE2M | -0.86919 |  | 7.311638 | -5.16316 | 4.64E-07 | 4.95E-06 | 5.83364 |
| TUBB6 | 0.648022 |  | 7.255472 | 5.154528 | 4.84E-07 | 5.13E-06 | 5.793463 |
| PVALB | -1.05953 |  | 6.504088 | -5.14854 | 4.98E-07 | 5.26E-06 | 5.765631 |
| RASGEF1C | -0.75739 |  | 5.905877 | -5.14339 | 5.11E-07 | 5.37E-06 | 5.741687 |
| CARD6 | 0.716297 |  | 6.129511 | 5.134797 | 5.33E-07 | 5.58E-06 | 5.701798 |
| NEUROD6 | -0.75362 |  | 7.379666 | -5.12929 | 5.47E-07 | 5.71E-06 | 5.676254 |
| SYT3 | -0.59176 |  | 7.485766 | -5.12917 | 5.48E-07 | 5.71E-06 | 5.675693 |
| MKRN3 | 0.585023 |  | 5.812975 | 5.12339 | 5.63E-07 | 5.85E-06 | 5.648937 |
| RHBDL1 | -0.71072 |  | 6.450977 | -5.12107 | 5.70E-07 | 5.91E-06 | 5.638179 |
| RAB27B | -0.62382 |  | 8.241302 | -5.11601 | 5.84E-07 | 6.02E-06 | 5.614805 |
| FAAH | -0.63511 |  | 7.413223 | -5.11282 | 5.93E-07 | 6.08E-06 | 5.600032 |
| C1orf87 | 0.73591 |  | 5.096875 | 5.111996 | 5.95E-07 | 6.10E-06 | 5.596236 |
| TXNIP | 0.63065 |  | 10.18217 | 5.100847 | 6.28E-07 | 6.40E-06 | 5.544758 |
| GRIA1 | -0.61304 |  | 8.032578 | -5.09124 | 6.58E-07 | 6.66E-06 | 5.50047 |
| RAI14 | 0.659927 |  | 7.061106 | 5.080587 | 6.93E-07 | 6.97E-06 | 5.451455 |
| SLC7A14 | -0.59117 |  | 7.75456 | -5.07682 | 7.05E-07 | 7.09E-06 | 5.434138 |
| CXCL1 | 0.688997 |  | 6.687126 | 5.069632 | 7.30E-07 | 7.32E-06 | 5.40113 |
| NXPH1 | -0.60847 |  | 7.30731 | -5.06945 | 7.31E-07 | 7.32E-06 | 5.400318 |
| RPP40 | -0.63269 |  | 7.600126 | -5.06565 | 7.44E-07 | 7.43E-06 | 5.382849 |
| ERC2 | -0.63458 |  | 9.311501 | -5.06017 | 7.64E-07 | 7.61E-06 | 5.35774 |
| KRTAP10-11 | 0.665257 |  | 5.666138 | 5.056284 | 7.78E-07 | 7.74E-06 | 5.339935 |
| IMPDH2 | -0.5875 |  | 8.871351 | -5.04828 | 8.09E-07 | 8.03E-06 | 5.303309 |
| NPTX2 | -0.62098 |  | 8.69661 | -5.04502 | 8.22E-07 | 8.13E-06 | 5.288379 |
| SYTL4 | 0.608643 |  | 6.596657 | 5.034834 | 8.63E-07 | 8.48E-06 | 5.241877 |
| KIF17 | -0.68935 |  | 6.37734 | -5.03409 | 8.66E-07 | 8.50E-06 | 5.23848 |
| CPNE6 | -0.64463 |  | 8.29254 | -5.03288 | 8.71E-07 | 8.53E-06 | 5.232966 |
| STAR | -0.66213 |  | 5.964916 | -5.02894 | 8.87E-07 | 8.68E-06 | 5.214975 |
| MPND | -0.58949 |  | 6.570352 | -5.01762 | 9.37E-07 | 9.11E-06 | 5.163409 |
| ST6GALNAC5 | -0.63633 |  | 8.039683 | -5.01385 | 9.54E-07 | 9.25E-06 | 5.146271 |
| CORT | -0.60662 |  | 6.862771 | -5.01231 | 9.61E-07 | 9.30E-06 | 5.139291 |
| MT1H | 0.66652 |  | 10.13295 | 5.012163 | 9.61E-07 | 9.30E-06 | 5.138609 |
| NUPR1 | 0.632336 |  | 7.621842 | 5.010108 | 9.71E-07 | 9.36E-06 | 5.129271 |
| SEMA5B | -0.72826 |  | 6.783932 | -5.00766 | 9.82E-07 | 9.46E-06 | 5.118134 |
| FGR | 0.719144 |  | 7.636286 | 4.99368 | 1.05E-06 | 1.01E-05 | 5.054708 |
| MTX2 | -0.60699 |  | 8.600166 | -4.99202 | 1.06E-06 | 1.01E-05 | 5.047165 |
| TAC1 | -0.86421 |  | 7.545354 | -4.99139 | 1.06E-06 | 1.02E-05 | 5.04435 |
| EMP3 | 0.654303 |  | 7.186938 | 4.968698 | 1.18E-06 | 1.12E-05 | 4.941716 |
| PYCRL | -0.75688 |  | 5.608149 | -4.96541 | 1.20E-06 | 1.13E-05 | 4.92686 |
| PAIP2B | 0.599959 |  | 7.730168 | 4.959686 | 1.23E-06 | 1.16E-05 | 4.901068 |
| CORO1A | -0.77707 |  | 7.802732 | -4.95056 | 1.29E-06 | 1.20E-05 | 4.85998 |
| P2RY14 | 0.742717 |  | 7.186815 | 4.941696 | 1.34E-06 | 1.25E-05 | 4.820115 |
| C14orf119 | -0.60476 |  | 7.102333 | -4.93094 | 1.41E-06 | 1.30E-05 | 4.771855 |
| SCN3B | -0.68638 |  | 9.633479 | -4.92921 | 1.42E-06 | 1.31E-05 | 4.764067 |
| TARBP1 | -0.67271 |  | 8.013009 | -4.9268 | 1.44E-06 | 1.32E-05 | 4.753281 |
| CYC1 | -0.60164 |  | 9.13833 | -4.91467 | 1.52E-06 | 1.39E-05 | 4.698979 |
| FGFR3 | 0.618999 |  | 10.19669 | 4.908686 | 1.57E-06 | 1.42E-05 | 4.672217 |
| C14orf79 | -0.61698 |  | 6.772983 | -4.90712 | 1.58E-06 | 1.43E-05 | 4.665213 |
| SYT1 | -0.74153 |  | 10.65565 | -4.90521 | 1.59E-06 | 1.44E-05 | 4.65671 |
| FSD1 | -0.63755 |  | 8.424312 | -4.90096 | 1.63E-06 | 1.47E-05 | 4.637697 |
| SHD | -0.6427 |  | 6.32784 | -4.8881 | 1.73E-06 | 1.55E-05 | 4.58039 |
| RGS4 | -0.64987 |  | 10.15819 | -4.88336 | 1.76E-06 | 1.58E-05 | 4.559303 |
| TNFRSF13C | 0.591499 |  | 5.692535 | 4.881728 | 1.78E-06 | 1.59E-05 | 4.552045 |
| MRPL37 | -0.67676 |  | 7.788712 | -4.87535 | 1.83E-06 | 1.63E-05 | 4.523713 |
| IGFBPL1 | -0.67185 |  | 6.688795 | -4.86878 | 1.89E-06 | 1.68E-05 | 4.494523 |
| SPTBN2 | -0.71958 |  | 6.603945 | -4.86786 | 1.90E-06 | 1.68E-05 | 4.490423 |
| ASNA1 | -0.67026 |  | 6.728876 | -4.84236 | 2.14E-06 | 1.87E-05 | 4.377565 |
| GOLT1A | -0.7636 |  | 4.751354 | -4.84158 | 2.14E-06 | 1.88E-05 | 4.374098 |
| CITED1 | -0.59617 |  | 6.604678 | -4.83999 | 2.16E-06 | 1.89E-05 | 4.367077 |
| NRSN2 | -0.60711 |  | 7.987884 | -4.83818 | 2.18E-06 | 1.90E-05 | 4.359079 |
| ADAMTS1 | 0.660657 |  | 7.404305 | 4.834303 | 2.22E-06 | 1.93E-05 | 4.341993 |
| PCDHAC2 | -0.6386 |  | 4.945053 | -4.83355 | 2.23E-06 | 1.94E-05 | 4.338653 |
| CACNA2D3 | -0.69816 |  | 8.833491 | -4.82677 | 2.30E-06 | 1.99E-05 | 4.308776 |
| PTPRT | -0.6761 |  | 7.590472 | -4.82089 | 2.36E-06 | 2.04E-05 | 4.282896 |
| PPFIA4 | -0.71143 |  | 8.795085 | -4.78835 | 2.74E-06 | 2.32E-05 | 4.140119 |
| LRRTM1 | -0.61201 |  | 7.459152 | -4.74622 | 3.33E-06 | 2.76E-05 | 3.956456 |
| CHAF1B | -0.59986 |  | 6.075602 | -4.70106 | 4.08E-06 | 3.33E-05 | 3.761115 |
| LRRC32 | 0.746147 |  | 6.777083 | 4.688583 | 4.32E-06 | 3.50E-05 | 3.707423 |
| ENDOG | -0.66854 |  | 7.664368 | -4.67622 | 4.57E-06 | 3.67E-05 | 3.654339 |
| LRRC4 | -0.67151 |  | 7.179659 | -4.67002 | 4.70E-06 | 3.75E-05 | 3.627762 |
| PPP1R14C | -0.6014 |  | 6.991099 | -4.66608 | 4.78E-06 | 3.81E-05 | 3.610916 |
| AKR1C3 | 0.587235 |  | 8.517787 | 4.663099 | 4.85E-06 | 3.86E-05 | 3.59814 |
| CCL2 | 0.732798 |  | 6.033794 | 4.627958 | 5.68E-06 | 4.46E-05 | 3.448283 |
| IRX3 | 0.68685 |  | 6.071896 | 4.621607 | 5.84E-06 | 4.57E-05 | 3.421301 |
| COG7 | -0.61909 |  | 6.513691 | -4.5867 | 6.83E-06 | 5.21E-05 | 3.273559 |
| CCDC81 | 0.630863 |  | 5.158202 | 4.553462 | 7.91E-06 | 5.90E-05 | 3.133802 |
| DLGAP3 | -0.60524 |  | 6.334369 | -4.53588 | 8.55E-06 | 6.34E-05 | 3.060223 |
| FCRLB | -0.69541 |  | 6.620887 | -4.53219 | 8.69E-06 | 6.43E-05 | 3.044821 |
| SLC39A10 | -0.64302 |  | 9.54766 | -4.49816 | 1.01E-05 | 7.35E-05 | 2.903185 |
| ENC1 | -0.67887 |  | 11.15582 | -4.4864 | 1.06E-05 | 7.70E-05 | 2.854439 |
| IFITM2 | 0.594405 |  | 10.61723 | 4.46511 | 1.17E-05 | 8.31E-05 | 2.766523 |
| LY6E | -0.64998 |  | 7.74572 | -4.44884 | 1.25E-05 | 8.84E-05 | 2.699555 |
| NEU1 | -0.60506 |  | 6.977154 | -4.43878 | 1.31E-05 | 9.18E-05 | 2.658279 |
| RANBP3L | 0.587105 |  | 5.197853 | 4.397074 | 1.57E-05 | 0.000107 | 2.487933 |
| SNAP91 | -0.612 |  | 11.3384 | -4.39652 | 1.57E-05 | 0.000108 | 2.485685 |
| TRIM56 | 0.705321 |  | 6.995583 | 4.389987 | 1.61E-05 | 0.00011 | 2.459129 |
| TRHDE | -0.65118 |  | 7.628531 | -4.38229 | 1.67E-05 | 0.000114 | 2.427891 |
| RFWD3 | -0.59978 |  | 5.814153 | -4.38111 | 1.68E-05 | 0.000114 | 2.423098 |
| TCIRG1 | 0.712123 |  | 6.637851 | 4.374243 | 1.73E-05 | 0.000117 | 2.39528 |
| DACH2 | -0.59585 |  | 7.962141 | -4.37036 | 1.76E-05 | 0.000119 | 2.379565 |
| KHDC1 | -0.60673 |  | 6.311619 | -4.36833 | 1.77E-05 | 0.00012 | 2.371338 |
| PLEKHA7 | 0.600922 |  | 6.043498 | 4.343427 | 1.97E-05 | 0.00013 | 2.270884 |
| UBE2T | -0.64646 |  | 6.515761 | -4.33014 | 2.08E-05 | 0.000137 | 2.217473 |
| NELL2 | -0.63293 |  | 11.58211 | -4.32384 | 2.14E-05 | 0.00014 | 2.192228 |
| UHRF1 | 0.625669 |  | 6.549978 | 4.306324 | 2.31E-05 | 0.00015 | 2.122128 |
| GIMAP7 | 0.66934 |  | 7.458241 | 4.296196 | 2.41E-05 | 0.000155 | 2.081714 |
| CCNA1 | -0.61401 |  | 6.72646 | -4.29127 | 2.46E-05 | 0.000157 | 2.062079 |
| BCAS1 | 0.659526 |  | 9.407165 | 4.286259 | 2.51E-05 | 0.000161 | 2.042144 |
| HIGD1B | 0.896655 |  | 6.833563 | 4.282203 | 2.55E-05 | 0.000163 | 2.026018 |
| RPA3 | -0.76196 |  | 6.860869 | -4.27821 | 2.60E-05 | 0.000165 | 2.010158 |
| PRR3 | -0.59146 |  | 6.189809 | -4.26663 | 2.73E-05 | 0.000172 | 1.964207 |
| PKN3 | 0.595346 |  | 6.006507 | 4.245672 | 2.98E-05 | 0.000186 | 1.881356 |
| TMEM169 | -0.58874 |  | 6.463666 | -4.20516 | 3.53E-05 | 0.000215 | 1.722186 |
| SLC30A3 | -0.68341 |  | 8.074216 | -4.19847 | 3.62E-05 | 0.000221 | 1.696047 |
| SCO1 | -0.61239 |  | 6.410946 | -4.163 | 4.20E-05 | 0.000251 | 1.557986 |
| SLC16A9 | 0.685496 |  | 7.886335 | 4.157616 | 4.29E-05 | 0.000256 | 1.537133 |
| MYH11 | 0.603132 |  | 6.575575 | 4.121686 | 4.97E-05 | 0.000291 | 1.398504 |
| CDC20 | -0.59841 |  | 5.767176 | -4.08739 | 5.72E-05 | 0.000327 | 1.267168 |
| MSH2 | -0.60367 |  | 6.552169 | -3.96585 | 9.32E-05 | 0.000502 | 0.809574 |
| TGFB1I1 | 0.735063 |  | 6.811238 | 3.957142 | 9.64E-05 | 0.000516 | 0.777269 |
| WDR18 | -0.61326 |  | 7.276641 | -3.95624 | 9.68E-05 | 0.000518 | 0.773927 |
| SUSD1 | -0.60338 |  | 6.619788 | -3.95085 | 9.89E-05 | 0.000528 | 0.753964 |
| GRP | -0.69932 |  | 6.055678 | -3.94955 | 9.94E-05 | 0.000529 | 0.749145 |
| IL4R | 0.611325 |  | 7.178121 | 3.925176 | 0.000109 | 0.000576 | 0.659196 |
| SLC47A2 | 0.612585 |  | 5.714473 | 3.925136 | 0.000109 | 0.000576 | 0.65905 |
| ASCC2 | -0.59027 |  | 6.890746 | -3.90805 | 0.000117 | 0.00061 | 0.596306 |
| PCDH8 | -0.60277 |  | 8.949506 | -3.88865 | 0.000126 | 0.000652 | 0.525313 |
| FAM71E1 | -0.60292 |  | 6.0395 | -3.86178 | 0.00014 | 0.000714 | 0.427573 |
| RPS4Y1 | -1.11484 |  | 7.792615 | -3.79632 | 0.00018 | 0.000891 | 0.191941 |
| SNCB | -0.65664 |  | 7.639409 | -3.7837 | 0.000189 | 0.000929 | 0.146914 |
| GALNTL5 | -0.6103 |  | 5.512211 | -3.6836 | 0.000276 | 0.001298 | -0.2053 |
| FLJ22184 | -0.63353 |  | 7.986382 | -3.67679 | 0.000284 | 0.001326 | -0.22895 |
| XIST | 1.238099 |  | 8.194638 | 3.541313 | 0.000467 | 0.00204 | -0.6912 |
| CDH13 | -0.59712 |  | 7.069193 | -3.47695 | 0.000589 | 0.002496 | -0.90527 |
| CFH | 0.65733 |  | 6.333081 | 3.450084 | 0.000648 | 0.002719 | -0.99356 |
| FOS | 0.592772 |  | 6.899512 | 3.143938 | 0.001848 | 0.006698 | -1.95501 |
